# Supplementary material for: Is there scope to improve the selection of patients with alcohol-related liver disease for referral to secondary care? A retrospective analysis of primary care referrals to a UK liver centre, incorporating simple blood tests
Source: BMJ Open. 2021 Jun 4;11(6):e047786. doi: 10.1136/bmjopen-2020-047786 (PMC8183275; doi:10.1136/bmjopen-2020-047786)
Supplement: Supplementary data [file bmjopen-2020-047786supp001.pdf]

Supplementary Table 1: Inclusion and exclusion criteria

Inclusion Criteria	Exclusion criteria
○ Age 18 or above	○ Presence of pre-existing hepatological diagnosis (Including but not limited to: auto-immune hepatitis, viral hepatitis, PBC, PSC, HCC),
○ Presence of new referral letter from GP to hepatology clinic at Royal Free during evaluation period Jan 2015 to Jan 2018	○ Patients are already under the care of a hepatologist/Gastroenterologist for investigation or management of a liver condition.
○ Primary reason for referral from GP to hepatologist is suspected diagnosis of ArLD$ or suspected diagnosis of NAFLD^	

$ ‘Suspected ArLD’ referrals were defined as those in which the GP referral letter requested an assessment by a liver specialist specifying concerns about suspected ArLD or expressing concerns about a patient’s alcohol intake.

^ ‘Suspected NAFLD’ referrals were defined as those in which the GP referral letter either specified that they were referring the patient to hepatology ‘with suspected NAFLD’ or ‘on the local NAFLD referral pathway’, OR, in the absence of any other cause of liver dysfunction, where the GP specified that the patient had steatosis or chronic liver disease on ultrasound in combination with mentioning metabolic risk factors (BMI ≥25, diabetes, high waist circumference, high cholesterol or hypertension).

Supplementary table 2: Demographics within each re-classified aetiology group

Overall characteristics (n=762)	ArLD (n =79)	NAFLD (n=451)	BAFLD (n=232)	
**Non advanced fibrosis (<F3) n (%)**	46/78 (60)	377/450 (83.8)	165/230 (71.7)	*p<0.001*
**Advanced fibrosis (≥F3) n (%)**	32/78 (40)	73/450 (16.2)	65/230 (28.3)	
**Age (mean; sd)**	51.85 ± 13.1	55.3 ± 14.07	57.2 ± 12.3	*p= 0.009*
**BMI (mean; sd)**	21.9 ± 2.32	32.1± 6.17	30.6 ± 5.03	*p<0.001*
**> 25 n (%)**	0/59 (0)	393/443 (88.7)	215/230 (93.3)	*p<0.001*
**> 30 n (%)**	0/57 (0)	252/416 (60.6)	98/202 (48.5)	*p<0.001*
**Alcohol intake median U/w (IQR)**	79.9 (49.3-140)	0 (0-4)	49.5 (30-88.5)	*p<0.001*
**N=**	76	434	228	
**Years of harmful drinking Median (IQR)**	13 (5-20)	0 (0-0)	20 (8-30)	*p<0.001*
**N=**	47	427	124	
**ALT median, (IQR)**	43 (28-68)	45 (31-68.25)	47 (30-67)	*p=0.752*
**N=**	79	n 450	232	
**Community ELF score (mean, sd)**	9.96 ± 0.42	10.33 ± 0.74	10.5 ± 0.84	*p= 0.215*
**N=**	7	169	54	
**Community FIB4 median (IQR)**	2.75 (1.22-5.19)	1.56 (1.38 -2.2)	2.2 (1.5-3.25)	*p =0.043*
**N=**	4	75	24	

ArLD = Alcohol-related Liver Disease, NAFLD = Non-Alcoholic-Fatty-Liver-Disease, BAFLD = Both Alcohol and Fatty Liver Disease, sd = standard deviation, BMI = Body Mass Index, IQR = interquartile range, ALT = alanine aminotransferase, ELF = Enhanced Liver Fibrosis score
