# Supplementary material for: Is there scope to improve the selection of patients with alcohol-related liver disease for referral to secondary care? A retrospective analysis of primary care referrals to a UK liver centre, incorporating simple blood tests
Source: BMJ Open. 2021 Jun 4;11(6):e047786. doi: 10.1136/bmjopen-2020-047786 (PMC8183275; doi:10.1136/bmjopen-2020-047786)
Supplement: Author's manuscript [file bmjopen-2020-047786.draft_revisions.pdf]

BMJ Open

BMJ Open is committed to open peer review. As part of this commitment we make the peer review history of every article we publish publicly available.

When an article is published we post the peer reviewers' comments and the authors' responses online. We also post the versions of the paper that were used during peer review. These are the versions that the peer review comments apply to.

The versions of the paper that follow are the versions that were submitted during the peer review process. They are not the versions of record or the final published versions. They should not be cited or distributed as the published version of this manuscript.

BMJ Open is an open access journal and the full, final, typeset and author-corrected version of record of the manuscript is available on our site with no access controls, subscription charges or pay-per-view fees (http://bmjopen.bmj.com).

If you have any questions on BMJ Open's open peer review process please email info.bmjopen@bmj.com

BMJ Open

A retrospective analysis of primary care referrals to secondary care with alcohol-related liver disease uncovers multimorbidity and need for stratification in the community

Journal:	*BMJ Open*
Manuscript ID	bmjopen-2020-047786
Article Type:	Original research
Date Submitted by the Author:	10-Dec-2020
Complete List of Authors:	Rhodes, Freya; UCL, Institute for Liver and Digestive Health, Division of Medicine, Royal Free Campus, Rowland Hill Street, NW3 2PF<br>Cococcia, Sara ; University of Pavia, First Department of Internal Medicine<br>Patel, Preya; UCL, Institute for Liver and Digestive Health, Division of Medicine, Royal Free Campus, Rowland Hill Street, NW3 2PF<br>Panovska-Griffiths, Jasmina; UCL, Department of Applied Health Research; UCL, Institute for Global Health<br>Tanwar, Sudeep; Barts Health NHS Trust, Department of Gastroenterology; UCL, Institute for Liver and Digestive Health, Division of Medicine, Royal Free Campus, Rowland Hill Street, NW3 2PF<br>Westbrook, R; UCL, Institute for Liver and Digestive Health, Division of Medicine, Royal Free Campus, Rowland Hill Street, NW3 2PF<br>Rodger, Alison; UCL, Department of Infection and Population Health<br>Rosenberg, William; UCL, Institute for Liver and Digestive Health, Division of Medicine, Royal Free Campus, Rowland Hill Street, NW3 2PF
Keywords:	Hepatology < INTERNAL MEDICINE, Organisation of health services < HEALTH SERVICES ADMINISTRATION & MANAGEMENT, PUBLIC HEALTH

SCHOLARONE™
Manuscripts

I, the Submitting Author has the right to grant and does grant on behalf of all authors of the Work (as defined in the below author licence), an exclusive licence and/or a non-exclusive licence for contributions from authors who are: i) UK Crown employees; ii) where BMJ has agreed a CC-BY licence shall apply, and/or iii) in accordance with the terms applicable for US Federal Government officers or employees acting as part of their official duties; on a worldwide, perpetual, irrevocable, royalty-free basis to BMJ Publishing Group Ltd ("BMJ") its licensees and where the relevant Journal is co-owned by BMJ to the co-owners of the Journal, to publish the Work in this journal and any other BMJ products and to exploit all rights, as set out in our licence.

The Submitting Author accepts and understands that any supply made under these terms is made by BMJ to the Submitting Author unless you are acting as an employee on behalf of your employer or a postgraduate student of an affiliated institution which is paying any applicable article publishing charge ("APC") for Open Access articles. Where the Submitting Author wishes to make the Work available on an Open Access basis (and intends to pay the relevant APC), the terms of reuse of such Open Access shall be governed by a Creative Commons licence – details of these licences and which Creative Commons licence will apply to this Work are set out in our licence referred to above.

Other than as permitted in any relevant BMJ Author's Self Archiving Policies, I confirm this Work has not been accepted for publication elsewhere, is not being considered for publication elsewhere and does not duplicate material already published. I confirm all authors consent to publication of this Work and authorise the granting of this licence.

For peer review only

A retrospective analysis of primary care referrals to secondary care with alcohol-related liver disease uncovers multimorbidity and need for stratification in the community

Rhodes F[1*], Cococcia S[1,2*], Patel, P[1], Panovska-Griffiths J[3,4], Tanwar S[5], Westbrook RH[1], Rodger A[4], Rosenberg WM[1]

[1]Institute for Liver and Digestive Health, UCL Division of Medicine, Royal Free Campus, London, UK
[2]First Department of Internal Medicine, San Matteo Hospital Foundation, University of Pavia, Pavia, Italy
[3]Department of Applied Health Research, University College London, UK
[4]Institute for Global Health, University College London, UK
[5] Bart's Health NHS Trust, London, United Kingdom

*joint first author

Author details:

Dr Freya Rhodes (Hepatology research fellow, Institute for Liver and Digestive health, UCL division of medicine, Royal Free Campus, London, UK NW3 2QG)
F.rhodes@doctors.net.uk

Dr Sara Cococcia (Gastroenterology registrar, Institute for Liver and Digestive health, UCL division of medicine, Royal Free Campus, London, UK NW3 2QG and First Department of Internal Medicine, San Matteo Hospital Foundation, University of Pavia, Pavia, Italy)

Dr Preya Patel (Gastroenterology registrar, Institute for Liver and Digestive health, UCL division of medicine, Royal Free Campus, London, UK NW3 2QG)

Dr Jasmina Panovska-Griffiths (Senior research associate in applied statistics, Department of Applied Health Research, University College London, UK and Institute for Global Health, University College London, UK)

Dr Sudeep Tanwar (Consultant Gastroenterologist & Hepatologist, Barts Health NHS Trust, and Honorary Associate Professor, UCL Institute for Liver & Digestive Health)

Dr Rachel Westbrook (Consultant hepatologist, Institute for Liver and Digestive health, UCL division of medicine, Royal Free Campus, London, UK NW3 2QG)

For peer review only

Prof Alison Rodger, Professor of infectious diseases, Institute for Global Health, University College London, UK

Prof William Rosenberg, Professor of Hepatology, Institute for Liver and Digestive health, UCL division of medicine, Royal Free Campus, London, UK NW3 2QG

Address for correspondence: w.rosenberg@ucl.ac.uk

Keywords: Liver fibrosis, Alcohol, NAFLD, referral pathway, non-invasive tests

Abbreviations:

ArLD: Alcohol related Liver Disease

CLD: Chronic Liver Disease

NAFLD: Non-Alcoholic Fatty Liver Disease

BAFLD: Both Alcohol and Fatty Liver Disease

AUD: Alcohol Use Disorder

NIT: Non-Invasive Test

ELF: Enhanced Liver Fibrosis

ALT: Alanine Aminotransferase

AST: Aspartate Aminotransferase

ALP: Alkaline Phosphatase

FIB4: Fibrosis 4 score

APRI: AST to Platelet Ratio Index

BMI: Body Mass Index

T2DM: Type II Diabetes Mellitus

OR: Odds Ratio

CI: Confidence Interval

GPs: General Practitioners

U/w: Units per week

Word count: 3,984, 2 figures, 2 tables

Author contributions: FR performed the analysis of the data, contributed to the collection of the data and wrote the first draft of the manuscript; SC contributed to the collection of data and to the writing of the manuscript; PP contributed to the review and editing of the manuscript, J P-G contributed to the statistical analysis. RW, ST and AR contributed to the analysis

For peer review only

methodology, and the review and editing of the manuscript. WR conceived the study, oversaw the study and reviewed and edited the manuscript drafts. All authors approved the final version of the paper. The corresponding author attests that all listed authors meet authorship criteria and that no others meeting the criteria have been omitted.

Funding

This study is being supported by funding from WMR's National Institute for Health Research Senior Investigator Award (Award number 200249). WMR is an NIHR Senior Investigator and is supported by the NIHR University College London Hospitals Biomedical Research Centre. JPG was supported by the United Kingdom National Institute for Health Research (NIHR) Applied Research Collaboration North Thames (ARC North Thames) at Bart's Health NHS Trust.

Competing interests statement:

WMR is an inventor of the ELF test but receives no related royalties. WMR has received speakers' fees from Siemens Healthineers. The other authors declare no competing interests.

Data sharing statement:

On publication of this article, the dataset will be made available from the corresponding author on reasonable request.

Transparency statement:

The lead author affirms that this manuscript is an honest, accurate and transparent account of the study being reported, and that no important aspects of the study have been omitted, nor discrepancies from the study originally planned.

Exclusive license statement:

The Corresponding Author has the right to grant on behalf of all authors and does grant on behalf of all authors, a worldwide licence to the Publishers and its licensees in perpetuity, in all forms, formats and media (whether known now or created in the future), to i) publish, reproduce, distribute, display and store the Contribution, ii) translate the Contribution into other languages, create adaptations, reprints, include within collections and create summaries, extracts and/or, abstracts of the Contribution, iii) create any other derivative work(s) based on the Contribution, iv) to exploit all subsidiary rights in the Contribution, v)

For peer review only - http://bmjopen.bmj.com/site/about/guidelines.xhtml

the inclusion of electronic links from the Contribution to third party material where-ever it may be located; and, vi) licence any third party to do any or all of the above."

Abstract:

Background:

Better outcomes of Alcohol-related Liver Disease (ArLD) and Non-Alcoholic-Fatty-Liver-Disease (NAFLD) depend on early detection of liver damage. Non-invasive tests (NIT) can improve case detection and reduce unnecessary referrals in NAFLD but their utility in ArLD and in patients with Both-Alcohol-and-Fatty-Liver-Disease (BAFLD) is unknown.

Objectives/outcome measures:

We aimed to determine the proportion of ArLD referrals with advanced fibrosis, the prevalence and demographics of BAFLD, and the potential impact of simple NIT on stratification.

Design/setting:

All new referrals from primary-care to a hepatology centre with suspected ArLD or NAFLD between Jan2015-Jan2018 were retrospectively reviewed. BAFLD was diagnosed in patients consuming >14 Units alcohol per week (U/w) and features of metabolic syndrome. Liver fibrosis severity was assessed using FibroScan, imaging, blood tests, clinical examination and liver histology where available.

Participants:

Of 2,944 new referrals, 762 (mean age 55.5±13.53 years) met inclusion criteria comprising 531 NAFLD and 231 ArLD, of which 147 (64%) could be reclassified as 'BAFLD'.

Results:

Amongst ArLD referrals 147/229 (64.2%) had no evidence of advanced fibrosis and were judged 'unnecessary'. Advanced fibrosis was observed in men drinking ≥50U/w (OR 2.74, 95% CI 1.51 to 5.00, p = 0.001), and ≥35U/w in women (OR 5.11, 95% CI 1.31 to 20.03, p = 0.019). Drinking > 14 U/w doubled the likelihood of advanced fibrosis in overweight/obesity (OR 2.11; CI 1.44 to 3.09; p<0.001). Use of FIB4 could halve unnecessary referrals (OR 0.50; CI 0.32 to 0.79, p = 0.003) with false negative rate of 22%, but was rarely used.

Conclusions:

The majority of referrals with suspected ArLD were deemed unnecessary. NIT could improve identification of liver damage in ArLD, BAFLD and NAFLD in primary-care. Anecdotal thresholds for harmful-drinking (35U/w in women and 50U/w in men) were validated. The impact of alcohol on NAFLD highlights the importance of multi-causality and multimorbidity in CLD.

For peer review only

Article Summary

Strengths and limitations of this study

- This is an evaluation of a single centre's referrals with ArLD investigating the interaction of obesity and alcohol as causes of liver disease.
- The study investigates the proportion of referred patients who could be managed more appropriately in primary care and evaluates for potential alcohol unit thresholds for harmful drinking.
- This was a retrospective study relying on data held in electronic clinical records, including of self-reported alcohol intake.
- This study lacked access to liver biopsy as a reference standard to stage fibrosis severity, but is reflective of 'real-world' clinical practice.

For peer review only

Introduction

It is estimated that 90% of all chronic liver disease (CLD) is preventable, with the commonest causes of cirrhosis attributed to ArLD and NAFLD (1).

Mortality from cirrhosis has increased 400% since 1970, and this is predominantly due to alcohol, although the rising prevalence of NAFLD is contributary (2). Whilst up to 90% of patients with the predisposing risk factors of Alcohol Use Disorder (AUD) or obesity will develop hepatic steatosis (3, 4), only approximately 20% of people with AUD (5) and 5% with NAFLD (6) will develop advanced fibrosis or cirrhosis. Both AUD and obesity can be managed effectively in primary-care but advanced fibrosis and cirrhosis warrant management in specialist liver centres. Detecting the minority of patients requiring specialist care is challenging because advanced fibrosis and most cases of cirrhosis are asymptomatic and simple liver blood tests (LFTs) and ultrasound imaging are neither sensitive nor specific in detecting advanced fibrosis or cirrhosis (7).

A direct consequence of the difficulty in detection of advanced fibrosis and cirrhosis is that three-quarters of people with CLD first present to healthcare when they already have advanced liver disease and the window for behaviour change or intervention to improve outcomes has often lapsed (1, 8, 9).

Conversely, people with suspected CLD are frequently referred to secondary-care when they do not have advanced fibrosis or cirrhosis and could have remained in primary-care for ongoing management. A previous study in 2015 found that 92% of NAFLD referrals were 'unnecessary', in that they had steatosis but no evidence of advanced fibrosis (10).

Subsequently a pathway involving a two-step diagnostic process incorporating Fibrosis-4 (FIB-4) and Enhanced Liver Fibrosis (ELF) NITs was successfully implemented in north central London in 2015 for people NAFLD, yielding an 88% reduction in 'unnecessary referrals' to hepatology with a five-fold increase in the detection of advanced fibrosis and cirrhosis, and significant cost-savings (10, 11). This study has influenced national guidelines on non-invasive testing in NAFLD (12). However, the proportion of referrals with alcohol use disorders (AUD) who do not have advanced ArLD that could be considered 'unnecessary' is unknown.

For peer review only - http://bmjopen.bmj.com/site/about/guidelines.xhtml

The ELF test has also been used successfully to triage patients from primary to secondary-care with alcohol use disorders in Denmark (13). The current UK national guidelines now recommend consideration of NIT in people with AUD in primary care, (7) however, the implementation of these pathways in the UK is not yet widespread and they are yet to be evaluated.

Whilst NAFLD and ALD have been described as distinct entities, many people live with risk factors for both conditions resulting in overlap between fat and alcohol as causes of CLD. Moreover, it is increasingly recognised that alcohol and fat are synergistic in causing liver damage, with obese people having increased risks of liver fibrosis for any given alcohol intake (7, 14-17). In this study, we have used the term 'BAFLD' (Both Alcohol and Fatty Liver Disease which was originally coined by the Parkes Group in Southampton) (18) to describe the combination of fat and alcohol as risk factors for CLD.

We aimed to determine the proportion of patients referred for investigation of ArLD from primary-care to secondary-care hepatology clinics that had evidence of advanced fibrosis; and the prevalence of both alcohol and fat as co-contributing factors. In addition, we aimed to determine the performance of simple NITs in the identification of cases of advanced fibrosis.

For peer review only

Methods

Study design

This is a retrospective cross-sectional analysis of all patients aged ≥18 years newly referred from primary-care to a hospital-based hepatology service at the Royal Free London NHS Foundation Trust (RFL), with a suspected diagnosis of Alcohol Related Liver Disease (ArLD) or Non-Alcoholic Fatty Liver Disease (NAFLD) between January 2015 and January 2018. Patients were excluded if they had any other hepatological diagnosis made prior to referral.

Study population

All referrals for ArLD or NAFLD during this period were reviewed in order to identify cases referred for NAFLD who were subsequently found to be drinking hazardous amounts of alcohol (>14 units per week). Sample size was based upon 3-years' worth of referrals. The primary outcome was the proportion of patients referred who were diagnosed with advanced fibrosis within 12 months from referral. Secondary outcomes included the primary reasons for referral, risk factors for developing advanced fibrosis, and the prevalence of BAFLD.

Data Collection

For peer review only - http://bmjopen.bmj.com/site/about/guidelines.xhtml

Anonymised data were extracted from the patients' electronic records. These included demographics, reason for referral, deprivation score, weight, height, waist circumference, alcohol intake, comorbidities, and any fibrosis assessment before and after referral. Where weight and height were unavailable, but clinical records reported that the patient was overweight or obese, they were categorised accordingly to BMI >25 (overweight) or BMI >30 (obese). FIB4 and APRI scores were calculated using the blood tests from the first attendance to clinic after referral.

The diagnosis of advanced fibrosis (equivalent to a histological stage of ≥ F3/4) or cirrhosis (≥ F4) was established by expert clinical judgement by hepatologists based on a composite of FibroScan, imaging, blood tests, clinical examination and liver histology where available, and this information was extracted from the electronic medical records. In the minority of cases where a diagnosis of advanced fibrosis was not clearly documented, decisions were reviewed by the study team (FR and SC) and consensus achieved. FibroScan was considered diagnostic for advanced fibrosis if the elasticity of a valid scan was ≥11kpa in ArLD (12, 19) and ≥10kpa in NAFLD patients (20).

In light of the frequent overlap between the two conditions, patients were subsequently recoded as having Both Alcohol and Fatty Liver Disease (BAFLD) if ArLD and NAFLD risk factors were both present. More specifically, BAFLD was applied to patients referred for suspected NAFLD who were subsequently found to be drinking more than 14 units of alcohol per week; and to patients who were referred for suspected ArLD, who also had either a BMI >25, or features of the metabolic syndrome. The metabolic syndrome was defined according to the International Diabetes Federation (IDF) and American Heart Association (AHA) as the presence of at least three of the following criteria: enlarged waist circumference (≥94cm in European men, ≥90cm South Asian men, ≥80cm women), hypercholesterolaemia, hypertension and type 2 diabetes (21).

Statistical analysis

Descriptive statistical analyses included calculations of the frequencies and percentages for categorical variables, while for continuous data means and standard deviation (SD) for normally distributed data, or medians and interquartile range (IQR) for skewed data were used. For the comparison of categorical variables, Chi-Squared or Fischer's exact test was used (the latter when n = <5), and for continuous data Mann Whitney-U or Student's-t test depending on the data distribution.

For peer review only

For peer review only - http://bmjopen.bmj.com/site/about/guidelines.xhtml

For data with more than three variables to compare, ANOVA or Kruskall Wallis ANOVA were used, depending on the distribution of the data.

Alcohol consumption was categorised into groups of units per week according to the perceived risk of liver damage established in the literature (7) (0-35, 36-50,51-100, >100 units per week) and into quartiles of the population distribution of alcohol consumption for the ArLD cohort in which few patients were drinking <50 units per week. Multiple binary logistic regression analysis was used to determine the association between key variables and the presence of advanced fibrosis. The key variables were those risk factors for fibrosis that were of established importance in the literature, and those associated with p values <0.25 in the univariate analysis. All p values were 2-sided and significance set at <0.05. All data were analysed using SPSS software (Version 25.0. Armonk, NY: IBM Corp), except for the odds ratios (ORs) for differences in outcomes for modelling of data with FIB4 compared with current practice, together with 95% confidence intervals and chi-square for statistical significance which were performed using MedCalc statistical software 2018.

Ethics

This study uses secondary anonymised patient data. The project was registered with the Integrated Research Application System (IRAS 272448) and judged to not require ethical approval using Health Research Authority guidance and to not require informed consent as it is a service evaluation. The study was reviewed by the Royal Free London NHS Foundation Trust Research and Development Office and registered with the audit and service evaluation department at the Royal Free Trust on 16th October 2019.

Patient and Public involvement

Patients and the Public were not involved in this study.

For peer review only

RESULTS

Patient demographics:

Between January 2015 and January 2018, a total of 2,944 patients were referred to the RFL hepatology service from primary care and of these, 762 (mean age 55.5±13.53 years) met the inclusion criteria for this study; 231 patients were referred with suspected ArLD (mean age 54.68±12.37 years), and 531 with suspected NAFLD (mean age 55.88±14 years). One patient was deemed to have active hepatitis C virus infection as comorbidity and three were found to have inactive chronic hepatitis B after referral. The demographic characteristics of the included patients are reported in **Table 1.** There was a higher proportion of male patients in the ArLD group (76.2%) than amongst the NAFLD group (54.2%, $p<0.001$). Active or previous smoking was significantly more common among those referred for ArLD compared to the NAFLD group (47.1% vs 11.3%; $p<0.001$). The average BMI was significantly higher in the NAFLD group than the ArLD group (31.9 and 27.9 kg/m^2 respectively, $p<0.001$), while median alcohol consumption was significantly higher in the ArLD group at 70 units/week (42-135), compared to 0 units/week (0-7) in the NAFLD group. The majority of the study population lay within the lowest 4 deciles of deprivation, and no significant difference in levels of deprivation was seen when ArLD and NAFLD referrals were compared (p=0.326).

For peer review only - http://bmjopen.bmj.com/site/about/guidelines.xhtml

Table 1: Baseline characteristics

Patient characteristics	Overall (n=762)	Suspected ArLD referrals* (n =231)	Suspected NAFLD referrals** (n=531)	
**Age** (mean; sd)	55.52 ±13.53	54.68±12.37	55.88±14	*p = 0.262*
Total n =	N = 762	N = 231	N = 531	
**Male** n (%)	464 (60.9%)	176 (76.2%)	288 (54.2%)	*p <0.001*
**Female** n (%)	298 (39.1%)	55 (23.8%)	243 (45.8%)	
Total n =	N = 762	N = 231	N = 531	
**BMI** (mean; sd)	30.85±6.23	27.9 ± 5.46 (n=174)	31.9 ±6.15	*p <0.001*
> 25 (n =)	608 (83.1%) n= 732	149 (70.6%) (n=211)	459 (88.1%) (n=521)	*p <0.001*
> 30 (n =)	350 (51.9%) n= 675	56 (30.3%) (n=185)	294 (60%) (n=490)	*p <0.001*
**Alcohol intake** U/w (median,IQR)	5, (0-42.75)	70 (42-134.8)	0 (0-7)	*p <0.001*
Total n =	N = 738	N = 226	N = 512	
**Years of harmful drinking** (median,IQR)	0 (0-3)	20 (6-30)	0 (0-0)	*p <0.001*
Total n =	N = 598	N = 143	N = 455	
**Diabetes**	235 (30.9%)	38 (16.5%)	197 (37.2%)	*p <0.001*
Total n =	N = 760	N = 231	N = 529	
**Hypertension**	397 (52.2%)	113 (48.9%)	284 (53.6%)	*p=0.236*
Total n =	N = 761	N = 231	N = 530	
**Hypercholesterolaemia**	352 (46.4%)	81 (35.1%)	271 (51.3%)	*p <0.001*
Total n =	N = 759	N = 231	N = 528	
**Smoking status:** Non- smoker n (%)	369 (54.2%)	65 (31.9%)	304 (63.7%)	*p<0.001*
Smoker n (%)	150 (22%)	96 (47.1)	54 (11.3%)	
Ex- smoker n (%)	162 (23.8%)	43 (21.1%)	119 (24.9%)	
Total n =	N = 681	N = 204	N = 477	
**ALT** (median,IQR)	45 (30-67)	47 (30-68)	45 (30-67)	*p =0.360*
Total n =	N= 761	N = 231	N = 530	
**Deprivation score rank** (median,IQR)	11314 (6451-17642)	10648 (6100-17464)	11637 (6578-17761)	*p =0.326*
**Deprivation score decile:** 1	51 (6.7%)	12 (5.2%)	39 (7.3%)	*p=0.264*
2	146 (25.9%)	53 (28.1%)	93 (24.9%)	
3	134 (43.4%)	42 (46.3%)	92 (42.2%)	
4	107 (57.5%)	30 (59.3%)	77 (56.7%)	
5	101 (70.7%)	33 (73.6%)	68 (69.5%)	
6	82 (81.5%)	26 (84.8%)	56 (80%)	
7	64 (89.9%)	17 (92.2%)	47 (88.9%)	
8	44 (95.7%)	8 (95.7%)	36 (95.7%)	
9	22 (98.6%)	6 (98.3%)	16 (98.7%)	
10	11 (100%)	4 (100%)	7 (100%)	

* Where primary reason for referral from GP was for suspected alcohol-related liver disease
**Where primary reason for referral from GP was for suspected NAFLD

For peer review only

Reasons for referral from primary care

The presence of hepatic steatosis on an ultrasound scan and abnormal LFTs were the commonest reasons for referral to hepatology clinic regardless of the aetiology. These were followed by elevated ELF and FIB4 in the NAFLD cohort (38.2 %and 16.9% respectively). Only 38/231 (16.4%) of patients with suspected ArLD had a NIT in primary-care prior to referral (25 ELF scores, 13 FIB4) and of these, 25/38 (66%) patients had comorbid features of the metabolic syndrome and so were subsequently recoded as BAFLD. Amongst the NAFLD referrals 293/531 (55.2%) had a NIT prior to referral in accordance with the local NAFLD pathway. Of these patients 203/293 (69%) were referred on the basis of an elevated ELF test and 90/293 (31%) based on their FIB4 score.

Risk of advanced fibrosis (>/F3) in patients referred with suspected ArLD.

Data on fibrosis stage were available for 758/762 patients following hepatology review, with four not attending for assessment. Of patients with suspected ArLD, 64.2% (147/229) had no evidence of advanced fibrosis and could be discharged back to primary care.

This figure was even higher in the NAFLD cohort with 83.4% not having advanced fibrosis. Univariate analysis of the 231 patients referred with ArLD revealed that advanced fibrosis was associated with raised ALP (OR 1.012, 95% CI 1.006 to 1.018 p <0.001) and higher alcohol consumption (alcohol data available for 224/231) (OR 1.006, 95% CI 1.002 to 1.010, p=0.006). When categorised into alcohol unit groups of: <35 U/w, 36-50 U/w, 51-100 U/w, >101 U/w; patients drinking >50 U/w had a higher risk of advanced fibrosis in this cohort (OR 2.899, 95% CI 1.068 to 7.869, p= 0.037). The multivariable logistic regression model found that the odds of advanced fibrosis in suspected ArLD was independently associated with increased units of alcohol consumed, (OR 1.007, 95%CI 1.002-1.012, p=0.007), ALP (OR 1.009, 95% CI 1.002-1.016, p=0.01), and reduced platelets (OR 0.992, 95%CI 0.988-0.996, p<0.001). There was a trend towards higher odds of advanced fibrosis with increased age, but this did not reach significance (p=0.059).

For peer review only - http://bmjopen.bmj.com/site/about/guidelines.xhtml

Patients with risk factors for both ArLD and NAFLD: 'BAFLD'.

Patients with risk factors for both ArLD and NAFLD were classified as BAFLD (as defined earlier) and the whole cohort was re-classified into three categories: ArLD, NAFLD and BAFLD, in order to evaluate further risk factors for advanced fibrosis **(figure 1).**

From the GP referral letters, 147 (63.6%) patients out of the 231 patients referred to the hepatology clinic with suspected ArLD were overweight, or met the diagnostic criteria of the metabolic syndrome and were therefore reclassified as BAFLD. Of the 531 patients referred to hepatology as suspected NAFLD, 80 of them (15.1%) also regularly consumed an average of more than 14 units per week and were reclassified as BAFLD. Overall, 83.1% of the whole cohort were overweight and 50% obese. As expected, the proportion of patients who were overweight and obese was significantly higher in the NAFLD cohort compared to ArLD cohort ($p<0.001$). The main characteristics of the three cohorts can be found in supplementary data.

Patients with BAFLD had almost double the prevalence of advanced fibrosis when compared to NAFLD (29% and 16.2% respectively, (OR 2.11, 95% CI 1.441 to 3.094), $p <0.001$, suggesting that hazardous drinking doubled the risk of fibrosis in people who are overweight or obese in this study population.

Patients in the ArLD cohort had the highest prevalence of advanced fibrosis (38%), and their weekly alcohol intake was almost double that of the BAFLD patients, precluding the opportunity to compare the impact of overweight/obesity on heavy alcohol consumption in this cohort.

Influence of alcohol on fibrosis risk

As the number of ArLD patients drinking <50 units per week (U/w) was small, the entire cohort (n=762) was examined in an attempt to identify a potential threshold for the effect of alcohol on fibrosis risk. Other factors influencing fibrosis risk including age and BMI were also studied. Alcohol data were available for 734/762 patients.

Increased alcohol U/w predicted advanced fibrosis (OR 1.009, 95%CI 1.006 to 1.012, $p = <0.001$) on univariate analysis.

Alcohol units were categorised into quartiles of the reported distribution of consumption (0-42 U/w, 43-70 U/w, 71-135 U/w, >136 U/w). Binary logistic regression revealed that patients consuming ≥43 U/w were at greater risk of advanced fibrosis than those drinking less than 43 U/w. (OR 1.814, 95%CI 1.038 to 3.172, $p = 0.037$), and those drinking ≥70 U/w were at

For peer review only - http://bmjopen.bmj.com/site/about/guidelines.xhtml

more than four times the risk of having advanced fibrosis compared with those drinking less than 43 U/w (OR 4.25, 95% CI 2.334 to 7.740, p = <0.001).

Alcohol consumption was then evaluated at literature-based unit thresholds of interest (0-35 U/w, 36-50 U/w, 51-100 U/w, >101 U/w) revealing that drinking more than 35 U/w was associated with double the odds of developing advanced fibrosis compared with those drinking <35 U/w (OR 2.173, 95% CI 1.119 to 4.219, p = 0.022) and the odds increased to over five-fold in those drinking more than 100 units per week (OR 5.044, 95% CI 3.071 to 8.284, p <0.001).

A different threshold effect was found when these data were analysed separately for men and women. In the overall cohort of 762 patients, the risk of having advanced fibrosis was higher in those men drinking >50 U/w (OR 2.743, 95% CI 1.506 to 4.998, p = 0.001), while in women the risk of having advanced fibrosis increased significantly at only >35 U/w (OR 5.115, 95% CI 1.306 to 20.030, p = 0.019), compared to <35 U/w).

In the overall cohort of 762 patients with ArLD/NAFLD/BAFLD (of which complete data for this model were available for 625/762), multivariable regression analysis revealed that increased units of alcohol, age, ALP, BMI and decreased platelet count were significantly associated with increased odds of a diagnosis of advanced fibrosis.

Modelling the impact of indirect fibrosis tests on the detection of advanced fibrosis in patients referred from primary care with suspected ArLD.

Blood test results from the first attendance at the secondary care were used to calculate FIB4 and APRI scores for 225/231 patients referred with suspected ArLD (6 patients did not have an AST value available). Median FIB4 and APRI were 1.58 (IQR 0.97-3.29) and 0.68 (IQR 0.36-1.53) respectively.

Both scores independently predicted the clinical diagnosis of advanced fibrosis in secondary care in multivariable regression analysis (for FIB4, OR=1.658, 95% CI 1.397 to 1.967, p <0.001; for APRI, OR=1.485, 95% CI 1.204 to 1.832, p <0.001).

When ROC analysis was used to examine the ability of NIT based on routine blood tests to predict a diagnosis of advanced fibrosis, FIB4 performed the best (AUROC 0.801), compared with APRI, AST, ALT, ALP and platelet count (All p <0.005 using DeLong comparison) and numerically but not significantly better than APRI (p = 0.06) **(figure 2).**

Amongst the cohort of patients with ArLD referred to secondary care, 35.81% were judged to have advanced fibrosis and thus 64.2% could be considered ‘unnecessary’ referrals. Use of a FIB4 threshold of ≥3.25 (22) could have improved the detection of patients with advanced

For peer review only - http://bmjopen.bmj.com/site/about/guidelines.xhtml

fibrosis nearly five-fold (OR=*4.82; 95% CI 2.56 to 9.09, p <0.0001)*, leading to a 79.3% reduction in unnecessary referrals to secondary care (64.2% to 27.1%) (OR = 0.21; 95% CI 0.11 to 0.39, p <0.001) However, this would be associated with the exclusion of 39 patients judged to have advanced fibrosis (false negative rate of 47.6%). **(Table 2).**

When modelling the referrals using a FIB4 threshold of ≥1.45,(22) the detection of advanced fibrosis improved two-fold compared with standard care (OR=1.98; 95% CI 1.27 to 3.09, p = 0.0027) and reduced the number of unnecessary referrals from 64.2% to 47.5% (OR=0.5; CI 0.32 to 0.79, p = 0.003), with 103 patients (45.7%) having a FIB4 score below 1.45 that could have remained in primary care. The false negative rate was lower using FIB4 ≥1.45compared to threshold ≥3.25 (18/103, 22% compared to 39/103, 47.5%; X^2=10.60; p=0.001).

Table 2: Accuracy of indirect fibrosis markers in detecting advanced fibrosis in a cohort of 231 patients referred from primary care with suspected ArLD. (N= 225/231.)

Indirect fibrosis test (n=225/231)	Correctly classifies	Sensitivity (95% CI)	Specificity (95% CI)	PPV (95% CI)	NPV (95% CI)	LR+ (95% CI)	LR- (95% CI)	TP FP	FN TN	False negative rate (%)	False Positive rate (%)
**APRI ≥ 1**	165 (73.3%)	64.6% (54-75)	78.3% (70-85)	63.1% (52-73)	80% (72-86)	3.02 (2.13-4.28)	0.44 (0.33-0.6)	53 31	29 112	35.4	21.7
**FIB4 ≥ 3.25**	170 (75.6%)	52.4% (41.2-63.5)	88.8% (82.2-93.3)	72.9% (59.5-83.3)	76.5% (69.1-82.6)	4.69 (2.83-7.77)	0.54 (0.43-0.67)	43 16	39 127	47.6	11.2
**FIB4 ≥1.45**	149 (66%)	78% (67.3-86.1)	59.4% (50.9-67.4)	52.4% (43.3-61.5)	82.5% (73.5-89)	1.92 (1.53-2.42)	0.37 (0.24-0.56)	64 58	18 85	22	40.6

Discussion

Two thirds of the patients referred to secondary care for suspected ArLD had no evidence of advanced fibrosis, representing unnecessary referrals. This can be explained in part because the commonest reasons for referral were abnormal LFTs and ultrasound scans, neither of which are sensitive or specific tests for advanced fibrosis (7). While some of these patients may have benefited from a hepatologist's advice about the wider consequences of their drinking, many primary care physicians consider that they are better placed to deliver brief advice about hazardous or harmful drinking and referral to liver specialists should be

For peer review only - http://bmjopen.bmj.com/site/about/guidelines.xhtml

For peer review only

restricted to patients with ArLD. Only 38/231 patients with suspected ArLD had any kind of fibrosis assessment prior to referral to secondary care, the majority of whom had features of metabolic syndrome or were overweight and received FIB4 and ELF tests suggesting that their GPs had followed the local NAFLD pathway that incorporates these investigations. These patients were reclassified as having BAFLD.

The majority (64%) of patients referred with suspected ArLD were overweight, obese or had features of metabolic syndrome. These patients with BAFLD had double the odds of advanced fibrosis when compared to the NAFLD cohort suggesting that hazardous drinking is associated with a doubling of the risk of liver fibrosis in people who are overweight or obese. This both highlights the increased risk of liver disease in patients with dual pathology and the importance of considering multimorbidity in chronic liver disease.

Although national guidelines state that the risk of advanced fibrosis develops at a lower alcohol unit threshold for women than men (<35 U/w for women, <50 U/w for men), (7) these thresholds are not based on published data that we have been able to identify. Few studies have investigated the association between levels of alcohol consumption and the risk of advanced fibrosis, and those that did have reported a range of thresholds (23-28). Furthermore, the levels of drinking that cause harm in the context of overweight and obesity are not known but we derived these same thresholds of 35 U/w in women and 50 U/w in this cohort of 762 patients that included a high prevalence of overweight and obese people. It should be noted that these thresholds focus purely on the risk of advanced liver fibrosis and cannot be generalized to other health measures. National guidelines state that there is an increased risk to health above 14 U/w.

The performance of "indirect" serum fibrosis tests is well reported in NAFLD, but less so in ArLD. In this study cohort of 231 ArLD patients, FIB4 and APRI outperformed simple liver blood tests (ALP, ALT, AST and platelet count) in predicting a diagnosis of advanced fibrosis on AUROC analysis, with FIB4 having the highest AUROC of 0.801. However, when examining FIB4 at literature-derived binary thresholds of 3.25 and 1.45, (13, 22) it did not perform as well in detecting clinically defined advanced fibrosis as has been reported in a recent study in which all participants were required to undergo liver biopsy (13). Stratifying patients in primary care using a FIB4 threshold of 3.25 could have reduced unnecessary referrals by 79.3%, with PPV and NPV for the detection of advanced fibrosis of 72.9% and 76.5% respectively. However, the associated false negative rate was 47.5% suggesting that nearly half the cases of advanced fibrosis would be left in primary care, making it unsuitable for case stratification. A FIB4 threshold of 1.45 produced a lesser, but still significant, false negative

For peer review only - http://bmjopen.bmj.com/site/about/guidelines.xhtml

rate of 22%, and although it reduced the proportion of unnecessary referrals by 50%, the PPV was 52.4% and overall, this threshold correctly classified only 66% of patients into presence or absence of advanced fibrosis. These results suggest that an effective ArLD pathway would require the use of either a NIT with better diagnostic performance or the use of two or more NIT in series, as employed in the Camden and Islington NAFLD pathway (10) .

This retrospective study lacked access to liver biopsy as a reference standard to stage fibrosis severity. Self-reported alcohol intake at the point of referral to secondary-care was used to record drinking behavior and this may not be reliable. However, this clinic-based sample of 'real-world' cases reflects current practice in the UK and many other countries and highlights the opportunity to stratify patients with ArLD community settings to ensure that only those with a high likelihood of advanced fibrosis are referred for liver specialist care.

Having so many 'unnecessary referrals' to secondary care is not only an inefficient use of resources, but also exposes patients to unnecessary investigation and the associated time, risk and anxiety. These patients could be managed more appropriately in community settings with an appropriate focus on the wider harms associated with their drinking. Conversely emphasis on those with advanced fibrosis might improve the early detection of those drinkers who are likely to progress to cirrhosis and suffer life limiting effects of their drinking.

Based on the performance of APRI and FIB4 in this cohort, we would not recommend their routine use to risk stratify patients with AUD. Instead, further evaluation of pathways incorporating non-invasive tests such as ELF or Fibroscan (7, 12) (13) would be preferable.

This study highlights the multi-causality and multi-morbidity endured by patients with ArLD and NAFLD. Although the interaction between alcohol and obesity is recognized, the low threshold of alcohol consumption at which the risk of advanced fibrosis nearly doubled in this cohort highlights the importance of communicating this risk to patients with fatty liver disease in clinics and through public health messaging. There is a need for greater awareness amongst healthcare professionals, policy makers and the public and a need for a multi-disciplinary approach to address the lifestyle risk factors that are likely to influence the morbidity and mortality of those with BAFLD.

In summary, the current referral strategy for patients with alcohol use disorders at risk of liver disease from primary care is inefficient and ineffective. There is a need for increased awareness of the need to search for fibrosis using appropriate strategies incorporating non-invasive testing, and education of the guidelines for fibrosis testing in both AUD and NAFLD. In addition, there is a need for improved collaboration between primary and secondary care services to develop referral pathways employing NIT, with evaluation to further refine

For peer review only - http://bmjopen.bmj.com/site/about/guidelines.xhtml

thresholds for referral and education to improve awareness and the advice provided to patient about the impact of overweight/obesity and alcohol on liver health.

What is known

- Majority of NAFLD referrals to secondary care do not have advanced fibrosis
- The addition of alcohol to a risk factor of obesity increases the risk of advanced fibrosis
- Patients with chronic liver disease, either ArLD or NAFLD related, often first present to healthcare when they have advanced liver disease

What this study adds

- 64.2% of alcohol referrals to secondary care are 'unnecessary'
- 64% of patients with AUD also had risk factors for NAFLD highlighting the importance of dual causality ('BAFLD')
- The alcohol unit threshold above which the risk of advanced fibrosis increase is 50 U/w in men, and 35 in women
- NIT were rarely used in primary care to select patients for referral to liver specialist
- While FIB4 could be used to halve the number of unnecessary alcohol referrals to liver specialists (at the expense of 22% false negative rate) the utility of more accurate fibrosis tests should be studied in alcohol referral pathways.

For peer review only - http://bmjopen.bmj.com/site/about/guidelines.xhtml

References

1. British-Liver-Trust. Alcohol Related Liver Disease: Statistics 2019 [Available from: https://britishlivertrust.org.uk/information-and-support/living-with-a-liver-condition/liver-conditions/alcohol/.
2. Williams R, Aspinall R, Bellis M, Camps-Walsh G, Cramp M, Dhawan A, et al. Addressing liver disease in the UK: a blueprint for attaining excellence in health care and reducing premature mortality from lifestyle issues of excess consumption of alcohol, obesity, and viral hepatitis. The Lancet. 2014;384(9958):1953-97.
3. Younossi ZM, Koenig AB, Abdelatif D, Fazel Y, Henry L, Wymer M. Global epidemiology of nonalcoholic fatty liver disease-Meta-analytic assessment of prevalence, incidence, and outcomes. Hepatology. 2016;64(1):73-84.
4. Gao B, Bataller R. Alcoholic liver disease: pathogenesis and new therapeutic targets. Gastroenterology. 2011;141(5):1572-85.
5. Teli MR, Day CP, Burt AD, Bennett MK, James OF. Determinants of progression to cirrhosis or fibrosis in pure alcoholic fatty liver. Lancet. 1995;346(8981):987-90.
6. Vernon G, Baranova A, Younossi ZM. Systematic review: the epidemiology and natural history of non-alcoholic fatty liver disease and non-alcoholic steatohepatitis in adults. Aliment Pharmacol Ther. 2011;34(3):274-85.
7. Newsome PN, Cramb R, Davison SM, Dillon JF, Foulerton M, Godfrey EM, et al. Guidelines on the management of abnormal liver blood tests. Gut. 2018;67(1):6-19.
8. Sheron N, Moore M, O'Brien W, Harris S, Roderick P. Feasibility of detection and intervention for alcohol-related liver disease in the community: the Alcohol and Liver Disease Detection study (ALDDeS). Br J Gen Pract. 2013;63(615):e698-705.
9. Hussain A, Patel PJ, Rhodes F, Srivastava A, Patch D, Rosenberg W. Decompensated cirrhosis is the commonest presentation for NAFLD patients undergoing liver transplant assessment. Clin Med (Lond). 2020;20(3):313-8.
10. Srivastava A, Gailer R, Tanwar S, Trembling P, Parkes J, Rodger A, et al. Prospective evaluation of a primary care referral pathway for patients with non-alcoholic fatty liver disease. J Hepatol. 2019;71(2):371-8.
11. Srivastava A, Jong S, Gola A, Gailer R, Morgan S, Sennett K, et al. Cost-comparison analysis of FIB-4, ELF and fibroscan in community pathways for non-alcoholic fatty liver disease. Bmc Gastroenterology. 2019;19.
12. NICE. Cirrhosis in Over 16s: Assessment and Management. National Institute for Health and Care Excellence: Guidance. 2016 [Available from: https://www.nice.org.uk.
13. Thiele M, Madsen BS, Hansen JF, Detlefsen S, Antonsen S, Krag A. Accuracy of the Enhanced Liver Fibrosis Test vs FibroTest, Elastography, and Indirect Markers in Detection of Advanced Fibrosis in Patients With Alcoholic Liver Disease. Gastroenterology. 2018;154(5):1369-79.
14. Ekstedt M, Franzen L, M H, Bendtsen P, Mathiesen UL, Bodemar G, et al. Alcohol consumption is associated with progression of hepatic fibrosis in non-alcoholic fatty liver disease. Scand J Gastroenterol. 2009;44(3):366-74.
15. Bataller R, Gao B. Liver fibrosis in alcoholic liver disease. Semin Liver Dis. 2015;35(2):146-56.
16. Becker U, Deis A, Sorensen TI, Gronbaek M, Borch-Johnsen K, Muller CF, et al. Prediction of risk of liver disease by alcohol intake, sex, and age: a prospective population study. Hepatology. 1996;23(5):1025-9.

For peer review only - http://bmjopen.bmj.com/site/about/guidelines.xhtml

17. Sanchez-Jimenez BA, Brizuela-Alcantara DC, Ramos-Ostos MH, Alva-lopez F, Uribe-Esquivel M, Chavez-Tapia N. Both alcoholic and non-alcoholic steatohepatitis association with cardiovascular risk and liver fibrosis. Alcohol. 2018;69:63-7.
18. Glyn-Owen K, Bohning D, Parkes J, Roderick P, Buchanan R. The combined effect of alcohol and obesity on risk of liver disease: a systematic review and meta-analysis. . Hepatology. 2019;70(S1):753A-A.
19. Nguyen-Khac E, Chatelain D, Tramier B, Decrombecque C, Robert B, Joly JP, et al. Assessment of asymptomatic liver fibrosis in alcoholic patients using fibroscan: Prospective comparison with seven non-invasive laboratory tests. Alimentary Pharmacology and Therapeutics. 2008;28(10):1188-98.
20. Chan WC, Treeprasertsuk S, Goh BBG, al. e. Optimising use of nonalcoholic fatty liver disease fibrosis score, Fibrosis-4 score, and liver stiffness measurement to identify patients with advanced fibrosis. Clin Gastroenterol Hepatol. 2019;17:2570-80.
21. Alberti KG, Eckel RH, Grundy SM, Zimmet PZ, Cleeman JI, Donato KA, et al. Harmonizing the metabolic syndrome: a joint interim statement of the International Diabetes Federation Task Force on Epidemiology and Prevention; National Heart, Lung, and Blood Institute; American Heart Association; World Heart Federation; International Atherosclerosis Society; and International Association for the Study of Obesity. Circulation. 2009;120(16):1640-5.
22. Moreno C, Mueller S, Szabo G. Non-invasive diagnosis and biomarkers in alcohol-related liver disease. J Hepatol. 2019;70(2):273-83.
23. Hagstrom H, Nasr P, Ekstedt M, Kechagias S, Onnerhag K, Nilsson E, et al. Low to moderate lifetime alcohol consumption is associated with less advanced stages of fibrosis in non-alcoholic fatty liver disease. Scandinavian Journal of Gastroenterology. 2017;52(2):159-65.
24. Corrao G, Bagnardi V, Zambon A, Arico S. Exploring the dose-response relationship between alcohol consumption and the risk of several alcohol-related conditions: a meta-analysis. Addiction. 1999;94(10):1551-73.
25. Corrao G, Bagnardi V, Zambon A, La Vecchia C. A meta-analysis of alcohol consumption and the risk of 15 diseases. Prev Med. 2004;38(5):613-9.
26. Corrao G, Bagnardi V, Zambon A, Torchio P. Meta-analysis of alcohol intake in relation to risk of liver cirrhosis. Alcohol Alcohol. 1998;33(4):381-92.
27. Kamper-Jorgensen M, Gronbaek M, Tolstrup J, Becker U. Alcohol and cirrhosis: dose--response or threshold effect? J Hepatol. 2004;41(1):25-30.
28. Patel PJ, Smith D, Connor JP, Horsfall LU, Hayward KL, Hossain F, et al. Alcohol Consumption in Diabetic Patients with Nonalcoholic Fatty Liver Disease. Can J Gastroenterol Hepatol. 2017;2017:7927685.

For peer review only

For peer review only - http://bmjopen.bmj.com/site/about/guidelines.xhtml

Figure 1: Flow chart depicting reclassification of aetiologies

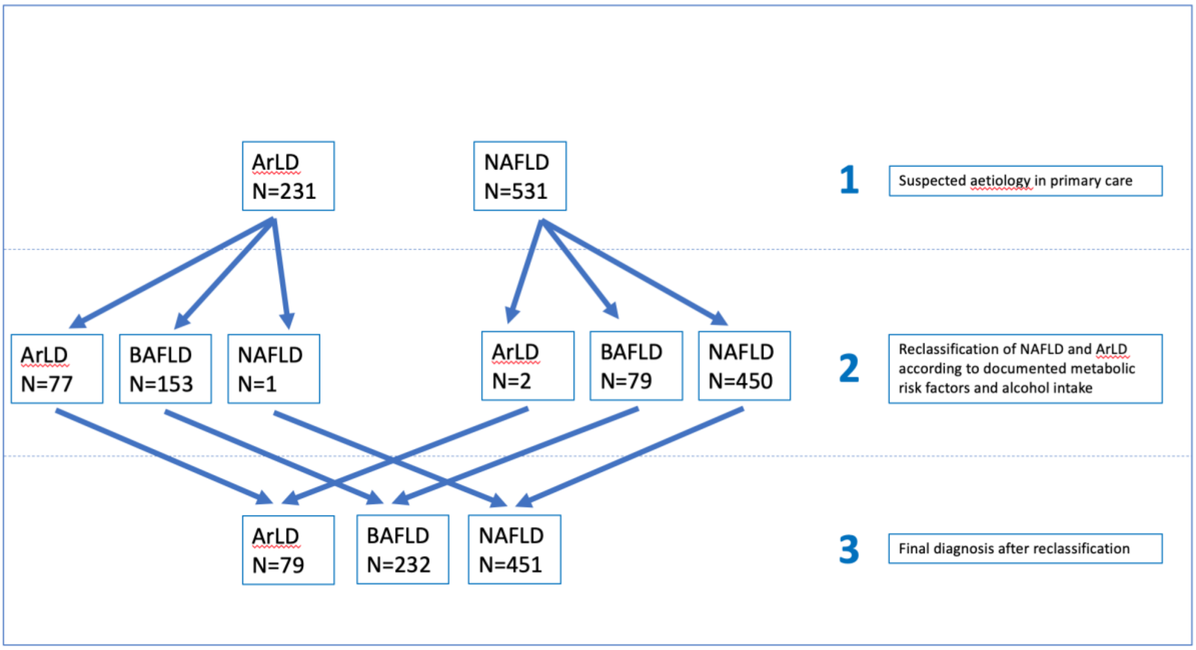

For peer review only

Figure 2: ROC analysis of the performance of indirect tests for fibrosis and simple liver blood tests in the detection of advanced fibrosis (composite clinical judgement) in patients referred with suspected ArLD. (N=231)

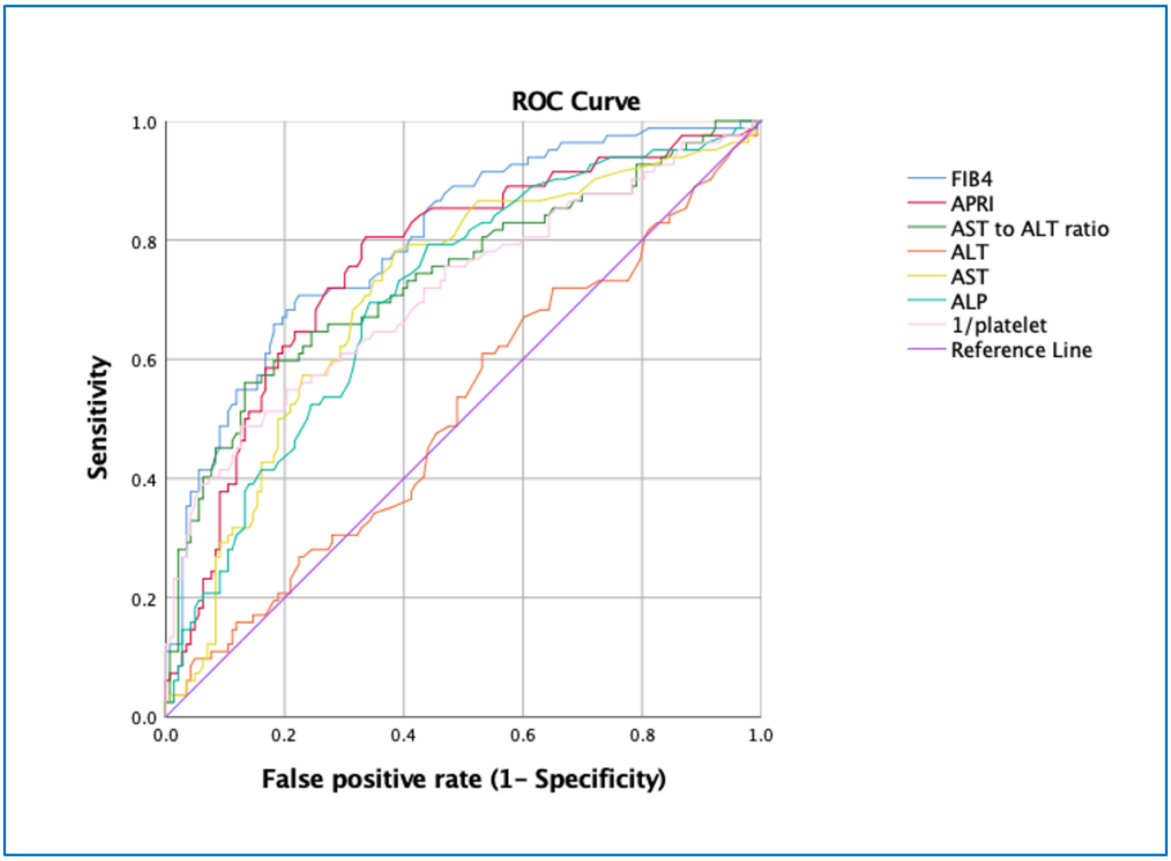

AUROCs with 95% CI in brackets: FIB4: 0.801 (0.742 to 0.860); APRI: 0.763 (0.697 to 0.829); AST:ALT ratio: 0.739 (0.668 to 0.809); ALT: 0.512 (0.433 to 0.591); AST: 0.711 (0.640 to 0.782); ALP: 0.708 (0.638 to 0.777); 1/platelet: 0.714 (0.641 to 0.787). (All p values <0.001 apart from ALT which was non-significant at p = 0.758)

Supplementary table 1: Demographics within each re-classified aetiology group

**Pt characteristics Overall (n=762)**	**ArLD (n =79)**	**NAFLD (n=451)**	**BAFLD* (n=232)**	
**Non advanced fibrosis** n (%) **(<F3)**	46/78 (60)	377/450 (83.8)	165/230 (71.7)	*p<0.001*
**Advanced fibrosis** n (%) **(F3 or above)**	32/78 (40)	73/450 (16.2)	65/230 (28.3)	
**Age** (mean; sd)	51.85±13.1	55.3±14.07	57.2±12.3	*p= 0.009*
Number	n = 79	n = 451	n = 232	
**Male** n (%)	55 (70)	220 (48.8)	189 (81.5)	*p<0.001*
**Female** n (%)	24 (30)	231 (51.2)	43 (18.5)	
Number	n = 79	n = 451	n = 232	
**BMI** (mean; sd)	21.9± 2.32	32.1±6.17	30.6 ±5.03	*p<0.001*
> 25 n (%)	0/59 (0)	393/443 (88.7)	215/230 (93.3)	*p<0.001*
> 30 n (%)	0/57 (0)	252/416 (60.6)	98/202 (48.5)	*p<0.001*
**Alcohol intake** U/w (median,IQR)	79.90 (49.3-140)	0 (0-4)	49.5 (30-88.5)	*p<0.001*
Number	n = 76	n = 434	n = 228	
**Years of harmful drinking** (median,IQR)	13 (5-20)	0 (0-0)	20 (8-30)	*p<0.001*
Number	n = 47	n = 427	n = 124	
**ALT** (median,IQR)	43 (28-68)	45 (31-68.25)	47 (30-67)	*p=0.752*
Number	n = 79	n = 450	n = 232	
**Community ELF score** (mean, sd)	9.96 ±0.42	10.33±0.74	10.5±0.84	*p= 0.215*
Number	n = 7	n = 169	n= 54	
**Community FIB4** (median,IQR)	2.75 (1.22-5.19)	1.56 (1.38 -2.2)	2.2 (1.5-3.25)	*p =0.043*
Number	n = 4	n= 75	n = 24	

STROBE Statement—Checklist of items that should be included in reports of ***cross-sectional studies***

	Item No	Recommendation	Page No
**Title and abstract**	1	(*a*) Indicate the study's design with a commonly used term in the title or the abstract	1
		(*b*) Provide in the abstract an informative and balanced summary of what was done and what was found	4
**Introduction**			
Background/rationale	2	Explain the scientific background and rationale for the investigation being reported	6
Objectives	3	State specific objectives, including any prespecified hypotheses	7
**Methods**			
Study design	4	Present key elements of study design early in the paper	7
Setting	5	Describe the setting, locations, and relevant dates, including periods of recruitment, exposure, follow-up, and data collection	7
Participants	6	(*a*) Give the eligibility criteria, and the sources and methods of selection of participants	7
Variables	7	Clearly define all outcomes, exposures, predictors, potential confounders, and effect modifiers. Give diagnostic criteria, if applicable	7
Data sources/ measurement	8*	For each variable of interest, give sources of data and details of methods of assessment (measurement). Describe comparability of assessment methods if there is more than one group	7
Bias	9	Describe any efforts to address potential sources of bias	7
Study size	10	Explain how the study size was arrived at	7
Quantitative variables	11	Explain how quantitative variables were handled in the analyses. If applicable, describe which groupings were chosen and why	8
Statistical methods	12	(*a*) Describe all statistical methods, including those used to control for confounding	8
		(*b*) Describe any methods used to examine subgroups and interactions	8
		(*c*) Explain how missing data were addressed	n/a
		(*d*) If applicable, describe analytical methods taking account of sampling strategy	n/a
		(*e*) Describe any sensitivity analyses	9
**Results**			
Participants	13*	(a) Report numbers of individuals at each stage of study—eg numbers potentially eligible, examined for eligibility, confirmed eligible, included in the study, completing follow-up, and analysed	10
		(b) Give reasons for non-participation at each stage	n/a
		(c) Consider use of a flow diagram	Fig1
Descriptive data	14*	(a) Give characteristics of study participants (eg demographic, clinical, social) and information on exposures and potential confounders	10
		(b) Indicate number of participants with missing data for each variable of interest	11
Outcome data	15*	Report numbers of outcome events or summary measures	12
Main results	16	(*a*) Give unadjusted estimates and, if applicable, confounder-adjusted estimates and their precision (eg, 95% confidence interval). Make clear which confounders were adjusted for and why they were included	12

For peer review only

For peer review only - http://bmjopen.bmj.com/site/about/guidelines.xhtml

		(*b*) Report category boundaries when continuous variables were categorized	13
		(*c*) If relevant, consider translating estimates of relative risk into absolute risk for a meaningful time period	n/a
Other analyses	17	Report other analyses done—eg analyses of subgroups and interactions, and sensitivity analyses	14
**Discussion**			
Key results	18	Summarise key results with reference to study objectives	15
Limitations	19	Discuss limitations of the study, taking into account sources of potential bias or imprecision. Discuss both direction and magnitude of any potential bias	17
Interpretation	20	Give a cautious overall interpretation of results considering objectives, limitations, multiplicity of analyses, results from similar studies, and other relevant evidence	17
Generalisability	21	Discuss the generalisability (external validity) of the study results	17
**Other information**			
Funding	22	Give the source of funding and the role of the funders for the present study and, if applicable, for the original study on which the present article is based	3

*Give information separately for exposed and unexposed groups.

Note: An Explanation and Elaboration article discusses each checklist item and gives methodological background and published examples of transparent reporting. The STROBE checklist is best used in conjunction with this article (freely available on the Web sites of PLoS Medicine at http://www.plosmedicine.org/, Annals of Internal Medicine at http://www.annals.org/, and Epidemiology at http://www.epidem.com/). Information on the STROBE Initiative is available at www.strobe-statement.org.

For peer review only

BMJ Open

Is there scope to improve the selection of patients with alcohol-related liver disease for referral to secondary care? A retrospective analysis of primary care referrals to a UK liver centre, incorporating simple blood tests.

Journal:	*BMJ Open*
Manuscript ID	bmjopen-2020-047786.R1
Article Type:	Original research
Date Submitted by the Author:	20-Feb-2021
Complete List of Authors:	Rhodes, Freya; UCL, Institute for Liver and Digestive Health, Division of Medicine, Royal Free Campus, Rowland Hill Street, NW3 2PF<br>Cococcia, Sara ; University of Pavia, First Department of Internal Medicine<br>Patel, Preya; UCL, Institute for Liver and Digestive Health, Division of Medicine, Royal Free Campus, Rowland Hill Street, NW3 2PF<br>Panovska-Griffiths, Jasmina; UCL, Department of Applied Health Research; UCL, Institute for Global Health<br>Tanwar, Sudeep; Barts Health NHS Trust, Department of Gastroenterology; UCL, Institute for Liver and Digestive Health, Division of Medicine, Royal Free Campus, Rowland Hill Street, NW3 2PF<br>Westbrook, R; UCL, Institute for Liver and Digestive Health, Division of Medicine, Royal Free Campus, Rowland Hill Street, NW3 2PF<br>Rodger, Alison; UCL, Department of Infection and Population Health<br>Rosenberg, William; UCL, Institute for Liver and Digestive Health, Division of Medicine, Royal Free Campus, Rowland Hill Street, NW3 2PF
**Primary Subject Heading**:	Gastroenterology and hepatology
Secondary Subject Heading:	General practice / Family practice, Public health
Keywords:	Hepatology < INTERNAL MEDICINE, Organisation of health services < HEALTH SERVICES ADMINISTRATION & MANAGEMENT, PUBLIC HEALTH

SCHOLARONE™
Manuscripts

I, the Submitting Author has the right to grant and does grant on behalf of all authors of the Work (as defined in the below author licence), an exclusive licence and/or a non-exclusive licence for contributions from authors who are: i) UK Crown employees; ii) where BMJ has agreed a CC-BY licence shall apply, and/or iii) in accordance with the terms applicable for US Federal Government officers or employees acting as part of their official duties; on a worldwide, perpetual, irrevocable, royalty-free basis to BMJ Publishing Group Ltd ("BMJ") its licensees and where the relevant Journal is co-owned by BMJ to the co-owners of the Journal, to publish the Work in this journal and any other BMJ products and to exploit all rights, as set out in our licence.

The Submitting Author accepts and understands that any supply made under these terms is made by BMJ to the Submitting Author unless you are acting as an employee on behalf of your employer or a postgraduate student of an affiliated institution which is paying any applicable article publishing charge ("APC") for Open Access articles. Where the Submitting Author wishes to make the Work available on an Open Access basis (and intends to pay the relevant APC), the terms of reuse of such Open Access shall be governed by a Creative Commons licence – details of these licences and which Creative Commons licence will apply to this Work are set out in our licence referred to above.

Other than as permitted in any relevant BMJ Author's Self Archiving Policies, I confirm this Work has not been accepted for publication elsewhere, is not being considered for publication elsewhere and does not duplicate material already published. I confirm all authors consent to publication of this Work and authorise the granting of this licence.

For peer review only

Is there scope to improve the selection of patients with alcohol-related liver disease for referral to secondary care? A retrospective analysis of primary care referrals to a UK liver centre, incorporating simple blood tests.

Rhodes F[1*], Cococcia S[1,2*], Patel, P[1], Panovska-Griffiths J[3,4], Tanwar S[5], Westbrook RH[1], Rodger A[4], Rosenberg WM[1]

[1]Institute for Liver and Digestive Health, UCL Division of Medicine, Royal Free Campus, London, UK
[2]First Department of Internal Medicine, San Matteo Hospital Foundation, University of Pavia, Pavia, Italy
[3]Department of Applied Health Research, University College London, UK
[4]Institute for Global Health, University College London, UK
[5] Bart's Health NHS Trust, London, United Kingdom

*joint first author

Author details:

Dr Freya Rhodes (Hepatology research fellow, Institute for Liver and Digestive health, UCL division of medicine, Royal Free Campus, London, UK NW3 2QG)
F.rhodes@doctors.net.uk

Dr Sara Cococcia (Gastroenterology registrar, Institute for Liver and Digestive health, UCL division of medicine, Royal Free Campus, London, UK NW3 2QG and First Department of Internal Medicine, San Matteo Hospital Foundation, University of Pavia, Pavia, Italy)

Dr Preya Patel (Gastroenterology registrar, Institute for Liver and Digestive health, UCL division of medicine, Royal Free Campus, London, UK NW3 2QG)

Dr Jasmina Panovska-Griffiths (Senior research associate in applied statistics, Department of Applied Health Research, University College London, UK and Institute for Global Health, University College London, UK)

Dr Sudeep Tanwar (Consultant Gastroenterologist & Hepatologist, Barts Health NHS Trust, and Honorary Associate Professor, UCL Institute for Liver & Digestive Health)

Dr Rachel Westbrook (Consultant hepatologist, Institute for Liver and Digestive health, UCL division of medicine, Royal Free Campus, London, UK NW3 2QG)

For peer review only - http://bmjopen.bmj.com/site/about/guidelines.xhtml

Prof Alison Rodger, Professor of infectious diseases, Institute for Global Health, University College London, UK

Prof William Rosenberg, Professor of Hepatology, Institute for Liver and Digestive health, UCL division of medicine, Royal Free Campus, London, UK NW3 2QG

Address for correspondence: w.rosenberg@ucl.ac.uk

Keywords: Liver fibrosis, Alcohol, NAFLD, referral pathway, non-invasive tests

Abbreviations:

ArLD: Alcohol related Liver Disease
CLD: Chronic Liver Disease
NAFLD: Non-Alcoholic Fatty Liver Disease
BAFLD: Both Alcohol and Fatty Liver Disease
AUD: Alcohol Use Disorder
NIT: Non-Invasive Test
ELF: Enhanced Liver Fibrosis
ALT: Alanine Aminotransferase
AST: Aspartate Aminotransferase
ALP: Alkaline Phosphatase
FIB4: Fibrosis 4 score
APRI: AST to Platelet Ratio Index
BMI: Body Mass Index
T2DM: Type II Diabetes Mellitus
OR: Odds Ratio
CI: Confidence Interval
GPs: General Practitioners
U/w: Units per week

Word count: 4,191, 2 figures, 2 tables

Author contributions: FR performed the analysis of the data, contributed to the collection of the data and wrote the first draft of the manuscript; SC contributed to the collection of data and to the writing of the manuscript; PP contributed to the review and editing of the manuscript, J P-G contributed to the statistical analysis. RW, ST and AR contributed to the analysis

For peer review only

methodology, and the review and editing of the manuscript. WR conceived the study, oversaw the study and reviewed and edited the manuscript drafts. All authors approved the final version of the paper. The corresponding author attests that all listed authors meet authorship criteria and that no others meeting the criteria have been omitted.

Funding

This study is being supported by funding from WMR's National Institute for Health Research Senior Investigator Award (Award number 200249). WMR is an NIHR Senior Investigator and is supported by the NIHR University College London Hospitals Biomedical Research Centre. JPG was supported by the United Kingdom National Institute for Health Research (NIHR) Applied Research Collaboration North Thames (ARC North Thames) at Bart's Health NHS Trust.

Competing interests statement:

WMR is an inventor of the ELF test but receives no related royalties. WMR has received speakers' fees from Siemens Healthineers. The other authors declare no competing interests.

Data sharing statement:

On publication of this article, the dataset will be made available from the corresponding author on reasonable request.

Transparency statement:

The lead author affirms that this manuscript is an honest, accurate and transparent account of the study being reported, and that no important aspects of the study have been omitted, nor discrepancies from the study originally planned.

Exclusive license statement:

The Corresponding Author has the right to grant on behalf of all authors and does grant on behalf of all authors, a worldwide licence to the Publishers and its licensees in perpetuity, in all forms, formats and media (whether known now or created in the future), to i) publish, reproduce, distribute, display and store the Contribution, ii) translate the Contribution into other languages, create adaptations, reprints, include within collections and create summaries, extracts and/or, abstracts of the Contribution, iii) create any other derivative work(s) based on the Contribution, iv) to exploit all subsidiary rights in the Contribution, v) the inclusion of electronic links from the Contribution to third party material where-ever it may be located; and, vi) licence any third party to do any or all of the above."

For peer review only - http://bmjopen.bmj.com/site/about/guidelines.xhtml

Abstract:

Objectives

Twenty-percent of people with alcohol-use-disorders develop advanced fibrosis and warrant referral to secondary-care. Improving outcomes in Alcohol-related-Liver-Disease (ArLD) relies on its earlier detection in primary-care with non-invasive-tests (NIT). We aimed to determine the proportion of alcohol-related referrals who were diagnosed with advanced fibrosis in secondary-care, the prevalence of 'BAFLD' (Both Alcohol and Fatty Liver Disease), and the potential impact of NIT on referral-stratification.

Design/setting

Retrospective analysis of all GP-referrals with suspected ArLD/NAFLD to a UK hepatology-centre between Jan2015-Jan2018.

Participants

Of 2,944 new referrals, 762 (mean age 55.5±13.53 years) met inclusion-criteria: 531 NAFLD and 231 ArLD, of which 147 (64%) could be reclassified as 'BAFLD'.

Primary outcome-measure: Proportion of referrals with suspected ArLD/NAFLD with advanced fibrosis as assessed by tertiary-centre hepatologists using combinations of FibroScan, imaging, examination and blood tests, and liver histology where indicated.

Secondary outcome-measures: Included impact of BMI/alcohol consumption on the odds of a diagnosis of advanced fibrosis, and performance of NIT in predicting advanced fibrosis in planned post-hoc analysis of referrals.

Results:

Amongst ArLD referrals 147/229 (64.2%) had no evidence of advanced fibrosis and were judged 'unnecessary'. Advanced fibrosis was observed in men drinking ≥50U/w (OR 2.74, 95% CI 1.51-to-5.00, p = 0.001), and ≥35U/w in women (OR 5.11, 95% CI 1.31-to-20.03, p = 0.019). Drinking > 14 U/w doubled the likelihood of advanced fibrosis in overweight/obesity (OR 2.11; CI 1.44-to-3.09; p<0.001). Use of FIB4 could halve unnecessary referrals (OR 0.50; CI 0.32-to-0.79, p = 0.003) with false-negative rate of 22%, but was rarely used.

Conclusions:

The majority of referrals with suspected ArLD were deemed unnecessary. NIT could improve identification of liver damage in ArLD, BAFLD and NAFLD in primary-care. Anecdotal thresholds for harmful-drinking (35U/w in women and 50U/w in men) were validated. The impact of alcohol on NAFLD highlights the importance of multi-causality in CLD.

For peer review only - http://bmjopen.bmj.com/site/about/guidelines.xhtml

Article Summary

Strengths and limitations of this study

- This study reflects real-world experience of consecutive alcohol referrals from primary care to a specialist liver centre over a 3-year period.
- Results of tests routinely performed in primary care can be used to improve selection of patients for referral.
- This was a retrospective study relying on data held in electronic clinical records, including of self-reported alcohol intake.
- Our study used consensus judgement of expert hepatologists to assess liver disease rather than liver biopsy as a reference standard to assess fibrosis severity, but is reflective of 'real-world' clinical practice.

For peer review only

Introduction

Approximately 90% of all chronic liver disease (CLD) is preventable, with the commonest causes of cirrhosis attributed to ArLD and NAFLD (1). Mortality from cirrhosis has increased 400% since 1970, predominantly due to alcohol, although the rising prevalence of NAFLD is contributary (2). Hepatic steatosis develops in up to 90% of people with Alcohol Use Disorder (AUD) or obesity (3, 4), but advanced fibrosis or cirrhosis will affect only approximately 20% of people with AUD (5) and 5% with NAFLD (6). Both AUD and obesity can be managed effectively in primary-care but advanced fibrosis and cirrhosis warrant management by liver specialists in secondary care. Detecting the minority of patients requiring specialist care is challenging because advanced fibrosis and most cases of cirrhosis are asymptomatic and simple liver blood tests (LFTs) and ultrasound imaging are neither sensitive nor specific in detecting advanced fibrosis or cirrhosis (7). As a consequence, three-quarters of people with CLD first present to healthcare with established advanced liver disease when behaviour change or therapeutic interventions have only modest impacts on prognosis (1, 8, 9).

Conversely, as many as 92% of people referred to secondary-care with suspected CLD do not have advanced fibrosis or cirrhosis requiring specialist care and could have remained in primary-care for ongoing management (10). Pathways of care employing the use of NITs for liver fibrosis (FIB-4) and the Enhanced Liver Fibrosis Test (ELF) in primary-care have been shown to be effective in the management of NAFLD, yielding an 88% reduction in 'unnecessary referrals' to liver specialists with a five-fold increase in the detection of advanced fibrosis and cirrhosis, and significant cost-savings (10, 11), influencing national guidelines(12). However, the proportion of referrals with AUD who do not have advanced ArLD that could be considered 'unnecessary' is unknown.

The ELF test has also been used successfully to triage patients from primary to secondary-care with AUD in Denmark (13). While current UK national guidelines recommend consideration of NIT in people with AUD in primary care (7), alcohol pathways employing NIT are not widely established in the UK and none have been evaluated to our knowledge.

Although NAFLD and ArLD are described as distinct entities for research purposes, the risk factors for both conditions co-exist in many patients. Moreover, it is increasingly recognised that alcohol and fat interact to cause liver damage, with obese people having increased risks of liver fibrosis for any given alcohol intake (7, 14-18). In this study we aimed to determine the proportion of patients referred for investigation of ArLD from primary-care to secondary-

For peer review only - http://bmjopen.bmj.com/site/about/guidelines.xhtml

care hepatology clinics that had evidence of advanced fibrosis; and the prevalence of both alcohol and fat as co-contributing factors to CLD, termed 'BAFLD' to describe the combination of Both Alcohol and Fatty Liver Disease (19). In addition, we aimed to determine the performance of simple NITs in the identification of cases of advanced fibrosis.

Methods

Study design

This is a retrospective cross-sectional analysis of consecutive patients aged ≥18 years newly referred from primary-care to a hospital-based hepatology service at the Royal Free London NHS Foundation Trust (RFL), with a suspected diagnosis of ArLD or NAFLD between January 2015 and January 2018. Patients were excluded if they had any other hepatological diagnosis made prior to referral.

Outcome measures

The primary outcome measure was the proportion of new patients referred from GP to hepatology clinic with suspected ArLD that had advanced fibrosis and could be deemed 'necessary' referrals.

Secondary outcome measures included the prevalence of 'BAFLD' amongst patients referred with suspected ArLD or NAFLD, analysis of demographic data as potential risk factors for a diagnosis of advanced fibrosis (including BMI, alcohol consumption, smoking status, age, sex, and deprivation score), and a post-hoc analysis of the performance of FIB4 and APRI in predicting a diagnosis of advanced fibrosis.

Study population

All electronic GP referrals for suspected ArLD or NAFLD during this period were reviewed in order to identify cases referred for NAFLD who were subsequently found to be drinking hazardous amounts of alcohol (>14 units per week). As these conditions were not always reliably coded and triaged from the outset, every new referral from GP to hepatology clinic during this time period was reviewed in order to select out the NAFLD and ArLD referrals to ensure cases were not missed. Sample size was based upon 3-years' worth of referrals.

'Suspected ArLD' referrals were defined as those in which the GP referral letter requested an assessment by a liver specialist specifying concerns about suspected ArLD or expressing concerns about a patient's alcohol intake.

'Suspected NAFLD' referrals were defined as those in which the GP referral letter either specified that they were referring the patient to hepatology 'with suspected NAFLD' or 'on

For peer review only - http://bmjopen.bmj.com/site/about/guidelines.xhtml

the local NAFLD referral pathway', OR, in the absence of any other cause of liver dysfunction, where the GP specified that the patient had steatosis or chronic liver disease on ultrasound in combination with mentioning metabolic risk factors (BMI ≥25, diabetes, high waist circumference, high cholesterol or hypertension).

Data Collection

Anonymised data were extracted from the patients' electronic records. These included demographics, reason for referral, deprivation score, weight, height, waist circumference, alcohol intake, comorbidities, and any fibrosis assessment before and after referral. Where weight and height were unavailable, but clinical records reported that the patient was overweight or obese, they were categorised accordingly to BMI >25 (overweight) or BMI >30 (obese). FIB4 and APRI scores were calculated using the blood tests from the first attendance to clinic after referral.

The diagnosis of advanced fibrosis (equivalent to a histological stage of ≥ F3/4) or cirrhosis (≥ F4) was established by expert clinical judgement by hepatologists based on a composite of FibroScan, imaging, blood tests, clinical examination and liver histology where available, and this information was extracted from the electronic medical records. In the minority of cases where a diagnosis of advanced fibrosis was not clearly documented, decisions were reviewed by the study team (FR and SC) and consensus achieved. FibroScan was considered diagnostic for advanced fibrosis if the elasticity of a valid scan was ≥11kpa in ArLD (12, 20) and ≥10kpa in NAFLD patients (21). For variables where any data were missing, the denominator used in the analysis was adjusted for only available data.

'Unnecessary referrals' were defined as those patients that, subsequent to an assessment by a liver specialist, were deemed not to have advanced fibrosis and could be discharged back to ongoing care in the community.

In light of the frequent overlap between the two conditions, patients were subsequently recoded as having Both Alcohol and Fatty Liver Disease (BAFLD) if ArLD and NAFLD risk factors were both present. More specifically, BAFLD was applied to patients referred for suspected NAFLD who were subsequently found to be drinking more than 14 units of alcohol per week; and to patients who were referred for suspected ArLD, who also had either a BMI >25, or features of the metabolic syndrome. The metabolic syndrome was defined according to the International Diabetes Federation (IDF) and American Heart Association (AHA) as the presence of at least three of the following criteria: enlarged waist circumference (≥94cm in

For peer review only - http://bmjopen.bmj.com/site/about/guidelines.xhtml

European men, ≥90cm South Asian men, ≥80cm women), hypercholesterolaemia, hypertension and type 2 diabetes (22).

Statistical analysis

Descriptive statistical analyses included calculations of the frequencies and percentages for categorical variables, while for continuous data means and standard deviation (SD) for normally distributed data, or medians and interquartile range (IQR) for skewed data were used. For the comparison of categorical variables, Chi-Squared or Fischer's exact test was used (the latter when n = <5), and for continuous data Mann Whitney-U or Student's-t test depending on the data distribution.

For data with more than three variables to compare, ANOVA or Kruskall Wallis ANOVA were used, depending on the distribution of the data.

Alcohol consumption was categorised into groups of units per week according to the perceived risk of liver damage established in the literature (7) (0-35, 36-50,51-100, >100 units per week) and into quartiles of the population distribution of alcohol consumption for the ArLD cohort in which few patients were drinking <50 units per week. Multiple binary logistic regression analysis was used to determine the association between key variables and the presence of advanced fibrosis. The key variables were those risk factors for fibrosis that were of established importance in the literature, and those associated with p values <0.25 in the univariate analysis. All p values were 2-sided and significance set at <0.05. All data were analysed using SPSS software (Version 25.0. Armonk, NY: IBM Corp), except for the odds ratios (ORs) for differences in outcomes for modelling of data with FIB4 compared with current practice, together with 95% confidence intervals and chi-square for statistical significance which were performed using MedCalc statistical software 2018.

Ethics

This study uses secondary anonymised patient data. The project was registered with the Integrated Research Application System (IRAS 272448) and judged to not require ethical approval or informed consent according to Health Research Authority guidance as it comprises data that were collected routinely as part of a registered service evaluation at the Royal Free London NHS Foundation Trust.

For peer review only

Patient and Public involvement

Patients and the Public were not involved in this study.

RESULTS

Patient demographics:

Between January 2015 and January 2018, a total of 2,944 patients were referred to the RFL hepatology service from primary care and of these, 762 (mean age 55.5±13.53 years) met the inclusion criteria for this study; 231 patients were referred with suspected ArLD (mean age 54.68±12.37 years), and 531 with suspected NAFLD (mean age 55.88±14 years). One patient was deemed to have active hepatitis C virus infection as comorbidity and three were found to have inactive chronic hepatitis B after referral. The demographic characteristics of the included patients are reported in **Table 1.** There was a higher proportion of male patients in the ArLD group (76.2%) than amongst the NAFLD group (54.2%, p<0.001). Active or previous smoking was significantly more common among those referred for ArLD compared to the NAFLD group (47.1% vs 11.3%; p<0.001). The average BMI was significantly higher in the NAFLD group than the ArLD group (31.9 and 27.9 kg/m^2 respectively, p<0.001), while median alcohol consumption was significantly higher in the ArLD group at 70 units/week (42-135), compared to 0 units/week (0-7) in the NAFLD group. The majority of the study population lay within the lowest 4 deciles of deprivation, and no significant difference in levels of deprivation was seen when ArLD and NAFLD referrals were compared (p=0.326).

For peer review only - http://bmjopen.bmj.com/site/about/guidelines.xhtml

Table 1: Baseline characteristics

Patient characteristics	Overall (n=762)	Suspected ArLD referrals* (n =231)	Suspected NAFLD referrals** (n=531)	
**Age** (mean; sd)	55.52 ±13.53	54.68±12.37	55.88±14	*p = 0.262*
**Male** n (%)	464 (60.9%)	176 (76.2%)	288 (54.2%)	*p <0.001*
**BMI** (mean; sd)	30.85 ± 6.23	27.9 ± 5.46 (n=174)	31.9 ±6.15	*p <0.001*
> 25 n (%)	608/732 (83.1)	149/211 (70.6)	459/521 (88.1)	*p <0.001*
> 30 n (%)	350/675 (51.9)	56/185 (30.3)	294/490 (60)	*p <0.001*
**Alcohol intake** U/w (median, IQR)	5, (0-42.75)	70 (42-134.8)	0 (0-7)	*p <0.001*
N =	738	226	512	
**Years of harmful drinking**				
Median (IQR)	0 (0-3)	20 (6-30)	0 (0-0)	*p <0.001*
Total n =	598	143	455	
**Diabetes** n (%)	235/760 (30.9)	38/231 (16.5)	197/529 (37.2)	*p <0.001*
**Hypertension** n (%)	397/761 (52.2)	113/231 (48.9)	284/530 (53.6)	*p=0.236*
**Hypercholesterolaemia** n (%)	352/759 (46.4)	81/231 (35.1)	271/528 (51.3)	*p <0.001*
**Smoking status:** Non- smoker n (%)	369/681 (54.2)	65/204 (31.9)	304/477 (63.7)	*p<0.001*
Smoker n (%)	150/681 (22)	96/204 (47.1)	54/477 (11.3)	
Ex- smoker n (%)	162/681 (23.8)	43/204 (21.1)	119/477 (24.9)	
**ALT** median (IQR)	45 (30-67)	47 (30-68)	45 (30-67)	*p =0.360*
N =	761	231	530	
**Deprivation score rank** Median (IQR)	11314 (6451-17642)	10648 (6100-17464)	11637 (6578-17761)	*p =0.326*
**Deprivation score decile**: 1	51 (6.7%)	12 (5.2%)	39 (7.3%)	*p=0.264*
2	146 (25.9%)	53 (28.1%)	93 (24.9%)	
3	134 (43.4%)	42 (46.3%)	92 (42.2%)	
4	107 (57.5%)	30 (59.3%)	77 (56.7%)	
5	101 (70.7%)	33 (73.6%)	68 (69.5%)	
6	82 (81.5%)	26 (84.8%)	56 (80%)	
7	64 (89.9%)	17 (92.2%)	47 (88.9%)	
8	44 (95.7%)	8 (95.7%)	36 (95.7%)	
9	22 (98.6%)	6 (98.3%)	16 (98.7%)	
10	11 (100%)	4 (100%)	7 (100%)	
**Had Biopsy** n (%)	122/762 (16%)	10/231 (4.3%)	112/531 (21.1.%)	*p <0.001*
**Had FibroScan** n (%)	575/762 (75.5%)	158/231(68.4%)	417/531 (78.5%)	*p =0.003*
**Valid FibroScan reading*****	524/575 (91%)	140/158 (89%)	389/417 (93%)	
**FibroScan** median Kpa (IQR)	5.5 (4.5-7.7)	6 (4.7-8.5)	5.4 (4.4-7.5)	*p = 0.03*

* Where primary reason for referral from GP was for suspected alcohol-related liver disease
**Where primary reason for referral from GP was for suspected NAFLD
*** FibroScan results were considered invalid if: IQR/M >30%, success rate <60%, <10 valid readings, or if this information was not recorded in the FibroScan report (missing information about IQR/M ratio/success rate made up n=22/575 FibroScan results).
SD = standard deviation, IQR = interquartile range, ALT = alanine aminotransferase, BMI = body mass index,

Reasons for referral from primary care

The presence of hepatic steatosis on an ultrasound scan and abnormal LFTs were the commonest reasons for referral to hepatology clinic regardless of the aetiology. These were followed by elevated ELF and FIB4 in the NAFLD cohort (38.2 %and 16.9% respectively).

For peer review only

For peer review only - http://bmjopen.bmj.com/site/about/guidelines.xhtml

Only 38/231 (16.4%) of patients with suspected ArLD had a NIT in primary-care prior to referral (25 ELF scores, 13 FIB4) and of these, 25/38 (66%) patients had comorbid features of the metabolic syndrome and so were subsequently recoded as BAFLD. Amongst the NAFLD referrals 293/531 (55.2%) had a NIT prior to referral in accordance with the local NAFLD pathway. Of these patients 203/293 (69%) were referred on the basis of an elevated ELF test and 90/293 (31%) based on their FIB4 score.

Prevalence of advanced fibrosis in patients referred with suspected ArLD or NAFLD.

Data on fibrosis stage were available for 758/762 patients following hepatology review, with four not attending for assessment. Of patients with suspected ArLD, 64.2% (147/229) had no evidence of advanced fibrosis and could be discharged back to primary-care. This figure was even higher in the NAFLD cohort with 83.4% not having advanced fibrosis.

Of the patients referred with suspected ArLD who had advanced fibrosis (82/229), the frequency with which fibrosis tests were used were: liver biopsy in 10% (8/82), FibroScan in 41% (34/82) and radiology in 62% (51/82).

Of the patients referred with suspected NAFLD who had advanced fibrosis (88/529), the frequency with which fibrosis tests were used were: liver biopsy in 47% (41/88), FibroScan in 64% (56/88) and radiology in 33% (29/88).

Risk of advanced fibrosis (>/F3) in patients referred with suspected ArLD.

Univariate analysis of the 231 patients referred with ArLD revealed that advanced fibrosis was associated with raised ALP (OR 1.012, 95% CI 1.006 to 1.018 p <0.001) and higher alcohol consumption (alcohol data available for 224/231) (OR 1.006, 95% CI 1.002 to 1.010, p=0.006). When categorised into alcohol unit groups of: <35 U/w, 36-50 U/w, 51-100 U/w, >101 U/w; patients drinking >50 U/w had a higher risk of advanced fibrosis in this cohort (OR 2.899, 95% CI 1.068 to 7.869, p= 0.037). The multivariable logistic regression model found that the odds of advanced fibrosis in suspected ArLD was independently associated with increased units of alcohol consumed, (OR 1.007, 95%CI 1.002-1.012, p=0.007), ALP (OR 1.009, 95% CI 1.002-1.016, p=0.01), and reduced platelets (OR 0.992, 95%CI 0.988-0.996, p<0.001). There was a trend towards higher odds of advanced fibrosis with increased age, but this did not reach significance (p=0.059).

For peer review only - http://bmjopen.bmj.com/site/about/guidelines.xhtml

Patients with risk factors for both ArLD and NAFLD: 'BAFLD'.

Patients with risk factors for both ArLD and NAFLD were classified as BAFLD (as defined earlier) and the whole cohort was re-classified into three categories: ArLD, NAFLD and BAFLD, in order to evaluate further risk factors for advanced fibrosis **(figure 1).**

From the GP referral letters, 147 (63.6%) patients out of the 231 patients referred to the hepatology clinic with suspected ArLD were overweight, or met the diagnostic criteria of the metabolic syndrome and were therefore reclassified as BAFLD. Of the 531 patients referred to hepatology as suspected NAFLD, 80 of them (15.1%) also regularly consumed an average of more than 14 units per week and were reclassified as BAFLD. Overall, 83.1% of the whole cohort were overweight and 50% obese. As expected, the proportion of patients who were overweight and obese was significantly higher in the NAFLD cohort compared to ArLD cohort ($p<0.001$). The main characteristics of the three cohorts can be found in supplementary data.

Patients with BAFLD had almost double the prevalence of advanced fibrosis when compared to NAFLD (29% and 16.2% respectively, (OR 2.11, 95% CI 1.441 to 3.094), $p <0.001$, suggesting that hazardous drinking doubled the risk of fibrosis in people who are overweight or obese in this study population.

Patients in the ArLD cohort had the highest prevalence of advanced fibrosis (38%), and their weekly alcohol intake was almost double that of the BAFLD patients, precluding the opportunity to compare the impact of overweight/obesity on heavy alcohol consumption in this cohort.

Influence of alcohol on fibrosis risk

As the number of ArLD patients drinking <50 units per week (U/w) was small, the entire cohort (n=762) was examined in an attempt to identify a potential threshold for the effect of alcohol on fibrosis risk. Other factors influencing fibrosis risk including age and BMI were also studied. Alcohol data were available for 734/762 patients.

Increased alcohol U/w predicted advanced fibrosis (OR 1.009, 95%CI 1.006 to 1.012, $p = <0.001$) on univariate analysis.

Alcohol units were categorised into quartiles of the reported distribution of consumption (0-42 U/w, 43-70 U/w, 71-135 U/w, >136 U/w). Binary logistic regression revealed that patients consuming ≥43 U/w were at greater risk of advanced fibrosis than those drinking less than 43 U/w. (OR 1.814, 95%CI 1.038 to 3.172, $p = 0.037$), and those drinking ≥70 U/w were at

For peer review only - http://bmjopen.bmj.com/site/about/guidelines.xhtml

more than four times the risk of having advanced fibrosis compared with those drinking less than 43 U/w (OR 4.25, 95% CI 2.334 to 7.740, p = <0.001).

Alcohol consumption was then evaluated at literature-based unit thresholds of interest (0-35 U/w, 36-50 U/w, 51-100 U/w, >101 U/w) revealing that drinking more than 35 U/w was associated with double the odds of developing advanced fibrosis compared with those drinking <35 U/w (OR 2.173, 95% CI 1.119 to 4.219, p = 0.022) and the odds increased to over five-fold in those drinking more than 100 units per week (OR 5.044, 95% CI 3.071 to 8.284, p <0.001).

A different threshold effect was found when these data were analysed separately for men and women. In the overall cohort of 762 patients, the risk of having advanced fibrosis was higher in those men drinking >50 U/w (OR 2.743, 95% CI 1.506 to 4.998, p = 0.001), while in women the risk of having advanced fibrosis increased significantly at only >35 U/w (OR 5.115, 95% CI 1.306 to 20.030, p = 0.019), compared to <35 U/w).

In the overall cohort of 762 patients with ArLD/NAFLD/BAFLD (of which complete data for this model were available for 625/762), multivariable regression analysis revealed that increased units of alcohol, age, ALP, BMI and decreased platelet count were significantly associated with increased odds of a diagnosis of advanced fibrosis.

Modelling the impact of indirect fibrosis tests on the detection of advanced fibrosis in patients referred from primary care with suspected ArLD.

Blood test results from the first attendance at the secondary care were used to calculate FIB4 and APRI scores for 225/231 patients referred with suspected ArLD (6 patients did not have an AST value available). Median FIB4 and APRI were 1.58 (IQR 0.97-3.29) and 0.68 (IQR 0.36-1.53) respectively.

Both scores independently predicted the clinical diagnosis of advanced fibrosis in secondary-care in multivariable regression analysis (for FIB4, OR=1.658, 95% CI 1.397 to 1.967, p <0.001; for APRI, OR=1.485, 95% CI 1.204 to 1.832, p <0.001).

When ROC analysis was used to examine the ability of NIT based on routine blood tests to predict a diagnosis of advanced fibrosis, FIB4 performed the best (AUROC 0.801), compared with APRI, AST, ALT, ALP and platelet count (All p <0.005 using DeLong comparison) and numerically but not significantly better than APRI (p = 0.06) **(figure 2).**

Amongst the cohort of patients with ArLD referred to secondary-care, 35.81% were judged to have advanced fibrosis and thus 64.2% could be considered 'unnecessary' referrals. Use of a FIB4 threshold of ≥3.25 (23) could have improved the detection of patients with advanced

For peer review only - http://bmjopen.bmj.com/site/about/guidelines.xhtml

fibrosis nearly five-fold (OR=*4.82; 95% CI 2.56 to 9.09, p <0.0001)*, leading to a 79.3% reduction in unnecessary referrals to secondary care (64.2% to 27.1%) (OR = 0.21; 95% CI 0.11 to 0.39, p <0.001) However, this would be associated with the exclusion of 39 patients judged to have advanced fibrosis (false negative rate of 47.6%). **(Table 2).**

When modelling the referrals using a FIB4 threshold of ≥1.45,(23) the detection of advanced fibrosis improved two-fold compared with standard-care (OR=1.98; 95% CI 1.27 to 3.09, p = 0.0027) and reduced the number of unnecessary referrals from 64.2% to 47.5% (OR=0.5; CI 0.32 to 0.79, p = 0.003), with 103 patients (45.7%) having a FIB4 score below 1.45 that could have remained in primary care. The false negative rate was lower using FIB4 ≥1.45compared to threshold ≥3.25 (18/103, 22% compared to 39/103, 47.5%; X^2=10.60; p=0.001).

Table 2: Accuracy of indirect fibrosis markers in detecting advanced fibrosis in a cohort of 231 patients referred from primary care with suspected ArLD. (N= 225/231.)

Indirect fibrosis test (n=225/231)	Correctly classifies	Sensitivity (95% CI)	Specificity (95% CI)	PPV (95% CI)	NPV (95% CI)	LR+ (95% CI)	LR- (95% CI)	TP FP	FN TN	False negative rate (%)	False Positive rate (%)
**APRI ≥ 1**	165 (73.3%)	64.6% (54-75)	78.3% (70-85)	63.1% (52-73)	80% (72-86)	3.02 (2.13-4.28)	0.44 (0.33-0.6)	53 31	29 112	35.4	21.7
**FIB4 ≥ 3.25**	170 (75.6%)	52.4% (41.2-63.5)	88.8% (82.2-93.3)	72.9% (59.5-83.3)	76.5% (69.1-82.6)	4.69 (2.83-7.77)	0.54 (0.43-0.67)	43 16	39 127	47.6	11.2
**FIB4 ≥1.45**	149 (66%)	78% (67.3-86.1)	59.4% (50.9-67.4)	52.4% (43.3-61.5)	82.5% (73.5-89)	1.92 (1.53-2.42)	0.37 (0.24-0.56)	64 58	18 85	22	40.6

Discussion

Two-thirds of the patients referred to secondary-care for suspected ArLD had no evidence of advanced fibrosis, representing unnecessary referrals. This can be explained in part because the commonest reasons for referral were abnormal LFTs and ultrasound scans, neither of which are sensitive or specific tests for advanced fibrosis (7). While some of these patients may have benefited from a hepatologist's advice about the wider consequences of their drinking, many primary-care physicians consider that they are better placed to deliver brief advice about hazardous or harmful drinking and referral to liver specialists should be

For peer review only - http://bmjopen.bmj.com/site/about/guidelines.xhtml

restricted to patients with ArLD. Only 38/231 patients with suspected ArLD had any kind of fibrosis assessment prior to referral to secondary-care, the majority of whom had features of metabolic syndrome or were overweight and received FIB4 and ELF tests suggesting that their GPs had followed the local NAFLD pathway that incorporates these investigations. These patients were reclassified as having BAFLD.

The majority (64%) of patients referred with suspected ArLD were overweight, obese or had features of metabolic syndrome. These patients with BAFLD had double the odds of advanced fibrosis when compared to the NAFLD cohort suggesting that hazardous drinking is associated with a doubling of the risk of liver fibrosis in people who are overweight or obese. This both highlights the increased risk of liver disease in patients with dual pathology and the importance of considering multimorbidity in chronic liver disease.

Although national guidelines state that the risk of advanced fibrosis develops at a lower alcohol unit threshold for women than men (<35 U/w for women, <50 U/w for men), (7) these thresholds are not based on published data that we have been able to identify. Few studies have investigated the association between levels of alcohol consumption and the risk of advanced fibrosis, and those that did have reported a range of thresholds (24-29). Furthermore, the levels of drinking that cause harm in the context of overweight and obesity are not known but we derived these same thresholds of 35 U/w in women and 50 U/w in this cohort of 762 patients that included a high prevalence of overweight and obese people. It should be noted that these thresholds focus purely on the risk of advanced liver fibrosis and cannot be generalized to other health measures. National guidelines state that there is an increased risk to health above 14 U/w.

The performance of "indirect" serum fibrosis tests is well reported in NAFLD, but less so in ArLD. In this study cohort of 231 ArLD patients, FIB4 and APRI outperformed simple liver blood tests (ALP, ALT, AST and platelet count) in predicting a diagnosis of advanced fibrosis on AUROC analysis, with FIB4 having the highest AUROC of 0.801. However, when examining FIB4 at literature-derived binary thresholds of 3.25 and 1.45, (13, 23) it did not perform as well in detecting clinically defined advanced fibrosis as has been reported in a recent study in which all participants were required to undergo liver biopsy (13). Stratifying patients in primary care using a FIB4 threshold of 3.25 could have reduced unnecessary referrals by 79.3%, with PPV and NPV for the detection of advanced fibrosis of 72.9% and 76.5% respectively. However, the associated false negative rate was 47.5% suggesting that nearly half the cases of advanced fibrosis would be left in primary care, making it unsuitable for case stratification. A FIB4 threshold of 1.45 produced a lesser, but still significant, false negative

For peer review only - http://bmjopen.bmj.com/site/about/guidelines.xhtml

rate of 22%, and although it reduced the proportion of unnecessary referrals by 50%, the PPV was 52.4% and overall, this threshold correctly classified only 66% of patients into presence or absence of advanced fibrosis. These results suggest that an effective ArLD pathway would require the use of either a NIT with better diagnostic performance or the use of two or more NIT in series, as employed in the Camden and Islington NAFLD pathway (10) .

This retrospective study lacked access to liver biopsy as a reference-standard to stage fibrosis severity. Self-reported alcohol intake at the point of referral to secondary-care was used to record drinking behavior and this may not be reliable. However, this clinic-based sample of 'real-world' cases reflects current practice in the UK and many other countries and highlights the opportunity to stratify patients with ArLD community settings to ensure that only those with a high likelihood of advanced fibrosis are referred for liver specialist care.

Having so many 'unnecessary referrals' to secondary-care is not only an inefficient use of resources, but also exposes patients to unnecessary investigation and the associated time, risk and anxiety. These patients could be managed more appropriately in community settings with an appropriate focus on the wider harms associated with their drinking. Conversely emphasis on those with advanced fibrosis might improve the early detection of those drinkers who are likely to progress to cirrhosis and suffer life-limiting effects of their drinking.

Based on the performance of APRI and FIB4 in this cohort, we would not recommend their routine use to risk stratify patients with AUD. Instead, further evaluation of pathways incorporating non-invasive tests such as ELF or FibroScan (7, 12) (13) would be preferable.

This study highlights the multi-causality and multi-morbidity endured by patients with ArLD and NAFLD. Although the interaction between alcohol and obesity is recognized, the low threshold of alcohol consumption at which the risk of advanced fibrosis nearly doubled in this cohort highlights the importance of communicating this risk to patients with fatty liver disease in clinics and through public health messaging. There is a need for greater awareness amongst healthcare professionals, policy makers and the public and a need for a multi-disciplinary approach to address the lifestyle risk factors that are likely to influence the morbidity and mortality of those with BAFLD.

In summary, the current referral strategy for patients with alcohol use disorders at risk of liver disease from primary care is inefficient and ineffective. There is a need for increased awareness of the need to search for fibrosis using appropriate strategies incorporating non-invasive testing, and education of the guidelines for fibrosis testing in both AUD and NAFLD. In addition, there is a need for improved collaboration between primary and secondary-care services to develop referral pathways employing NIT, with evaluation to further refine

For peer review only

thresholds for referral and education to improve awareness and the advice provided to patient about the impact of overweight/obesity and alcohol on liver health.

For peer review only

References

For peer review only - http://bmjopen.bmj.com/site/about/guidelines.xhtml

1. British-Liver-Trust. Alcohol Related Liver Disease: Statistics 2019 [Available from: https://britishlivertrust.org.uk/information-and-support/living-with-a-liver-condition/liver-conditions/alcohol/.]

2. Williams R, Aspinall R, Bellis M, Camps-Walsh G, Cramp M, Dhawan A, et al. Addressing liver disease in the UK: a blueprint for attaining excellence in health care and reducing premature mortality from lifestyle issues of excess consumption of alcohol, obesity, and viral hepatitis. The Lancet. 2014;384(9958):1953-97.

3. Younossi ZM, Koenig AB, Abdelatif D, Fazel Y, Henry L, Wymer M. Global epidemiology of nonalcoholic fatty liver disease-Meta-analytic assessment of prevalence, incidence, and outcomes. Hepatology. 2016;64(1):73-84.

4. Gao B, Bataller R. Alcoholic liver disease: pathogenesis and new therapeutic targets. Gastroenterology. 2011;141(5):1572-85.

5. Teli MR, Day CP, Burt AD, Bennett MK, James OF. Determinants of progression to cirrhosis or fibrosis in pure alcoholic fatty liver. Lancet. 1995;346(8981):987-90.

6. Vernon G, Baranova A, Younossi ZM. Systematic review: the epidemiology and natural history of non-alcoholic fatty liver disease and non-alcoholic steatohepatitis in adults. Aliment Pharmacol Ther. 2011;34(3):274-85.

7. Newsome PN, Cramb R, Davison SM, Dillon JF, Foulerton M, Godfrey EM, et al. Guidelines on the management of abnormal liver blood tests. Gut. 2018;67(1):6-19.

8. Sheron N, Moore M, O'Brien W, Harris S, Roderick P. Feasibility of detection and intervention for alcohol-related liver disease in the community: the Alcohol and Liver Disease Detection study (ALDDeS). Br J Gen Pract. 2013;63(615):e698-705.

9. Hussain A, Patel PJ, Rhodes F, Srivastava A, Patch D, Rosenberg W. Decompensated cirrhosis is the commonest presentation for NAFLD patients undergoing liver transplant assessment. Clin Med (Lond). 2020;20(3):313-8.

10. Srivastava A, Gailer R, Tanwar S, Trembling P, Parkes J, Rodger A, et al. Prospective evaluation of a primary care referral pathway for patients with non-alcoholic fatty liver disease. J Hepatol. 2019;71(2):371-8.

11. Srivastava A, Jong S, Gola A, Gailer R, Morgan S, Sennett K, et al. Cost-comparison analysis of FIB-4, ELF and fibroscan in community pathways for non-alcoholic fatty liver disease. Bmc Gastroenterology. 2019;19.

12. NICE-Guidance. Cirrhosis in Over 16s: Assessment and Management. National Institute for Health and Care Excellence: Guidance. 2016 [Available from: https://www.nice.org.uk.]

13. Thiele M, Madsen BS, Hansen JF, Detlefsen S, Antonsen S, Krag A. Accuracy of the Enhanced Liver Fibrosis Test vs FibroTest, Elastography, and Indirect Markers in Detection of Advanced Fibrosis in Patients With Alcoholic Liver Disease. Gastroenterology. 2018;154(5):1369-79.

14. Ekstedt M, Franzen L, M H, Bendtsen P, Mathiesen UL, Bodemar G, et al. Alcohol consumption is associated with progression of hepatic fibrosis in non-alcoholic fatty liver disease. Scand J Gastroenterol. 2009;44(3):366-74.

15. Bataller R, Gao B. Liver fibrosis in alcoholic liver disease. Semin Liver Dis. 2015;35(2):146-56.

16. Becker U, Deis A, Sorensen TI, Gronbaek M, Borch-Johnsen K, Muller CF, et al. Prediction of risk of liver disease by alcohol intake, sex, and age: a prospective population study. Hepatology. 1996;23(5):1025-9.

For peer review only - http://bmjopen.bmj.com/site/about/guidelines.xhtml

17. Sanchez-Jimenez BA, Brizuela-Alcantara DC, Ramos-Ostos MH, Alva-lopez F, Uribe-Esquivel M, Chavez-Tapia N. Both alcoholic and non-alcoholic steatohepatitis association with cardiovascular risk and liver fibrosis. Alcohol. 2018;69:63-7.
18. Glyn-Owen K, Bohning D, Parkes J, Roderick P, Buchanan R. The combined effect of alcohol and body mass index on risk of chronic liver disease: A systematic review and meta-analysis of cohort studies. Liver Int. 2020.
19. Glyn-Owen K, Bohning D, Parkes J, Roderick P, Buchanan R. The combined effect of alcohol and obesity on risk of liver disease: a systematic review and meta-analysis. . Hepatology. 2019;70(S1):753A-A.
20. Nguyen-Khac E, Chatelain D, Tramier B, Decrombecque C, Robert B, Joly JP, et al. Assessment of asymptomatic liver fibrosis in alcoholic patients using fibroscan: Prospective comparison with seven non-invasive laboratory tests. Alimentary Pharmacology and Therapeutics. 2008;28(10):1188-98.
21. Chan WC, Treeprasertsuk S, Goh BBG, al. e. Optimising use of nonalcoholic fatty liver disease fibrosis score, Fibrosis-4 score, and liver stiffness measurement to identify patients with advanced fibrosis. Clin Gastroenterol Hepatol. 2019;17:2570-80.
22. Alberti KG, Eckel RH, Grundy SM, Zimmet PZ, Cleeman JI, Donato KA, et al. Harmonizing the metabolic syndrome: a joint interim statement of the International Diabetes Federation Task Force on Epidemiology and Prevention; National Heart, Lung, and Blood Institute; American Heart Association; World Heart Federation; International Atherosclerosis Society; and International Association for the Study of Obesity. Circulation. 2009;120(16):1640-5.
23. Moreno C, Mueller S, Szabo G. Non-invasive diagnosis and biomarkers in alcohol-related liver disease. J Hepatol. 2019;70(2):273-83.
24. Hagstrom H, Nasr P, Ekstedt M, Kechagias S, Onnerhag K, Nilsson E, et al. Low to moderate lifetime alcohol consumption is associated with less advanced stages of fibrosis in non-alcoholic fatty liver disease. Scandinavian Journal of Gastroenterology. 2017;52(2):159-65.
25. Corrao G, Bagnardi V, Zambon A, Arico S. Exploring the dose-response relationship between alcohol consumption and the risk of several alcohol-related conditions: a meta-analysis. Addiction. 1999;94(10):1551-73.
26. Corrao G, Bagnardi V, Zambon A, La Vecchia C. A meta-analysis of alcohol consumption and the risk of 15 diseases. Prev Med. 2004;38(5):613-9.
27. Corrao G, Bagnardi V, Zambon A, Torchio P. Meta-analysis of alcohol intake in relation to risk of liver cirrhosis. Alcohol Alcohol. 1998;33(4):381-92.
28. Kamper-Jorgensen M, Gronbaek M, Tolstrup J, Becker U. Alcohol and cirrhosis: dose--response or threshold effect? J Hepatol. 2004;41(1):25-30.
29. Patel PJ, Smith D, Connor JP, Horsfall LU, Hayward KL, Hossain F, et al. Alcohol Consumption in Diabetic Patients with Nonalcoholic Fatty Liver Disease. Can J Gastroenterol Hepatol. 2017;2017:7927685.

For peer review only - http://bmjopen.bmj.com/site/about/guidelines.xhtml

Figure 1: Flow chart depicting reclassification of aetiologies

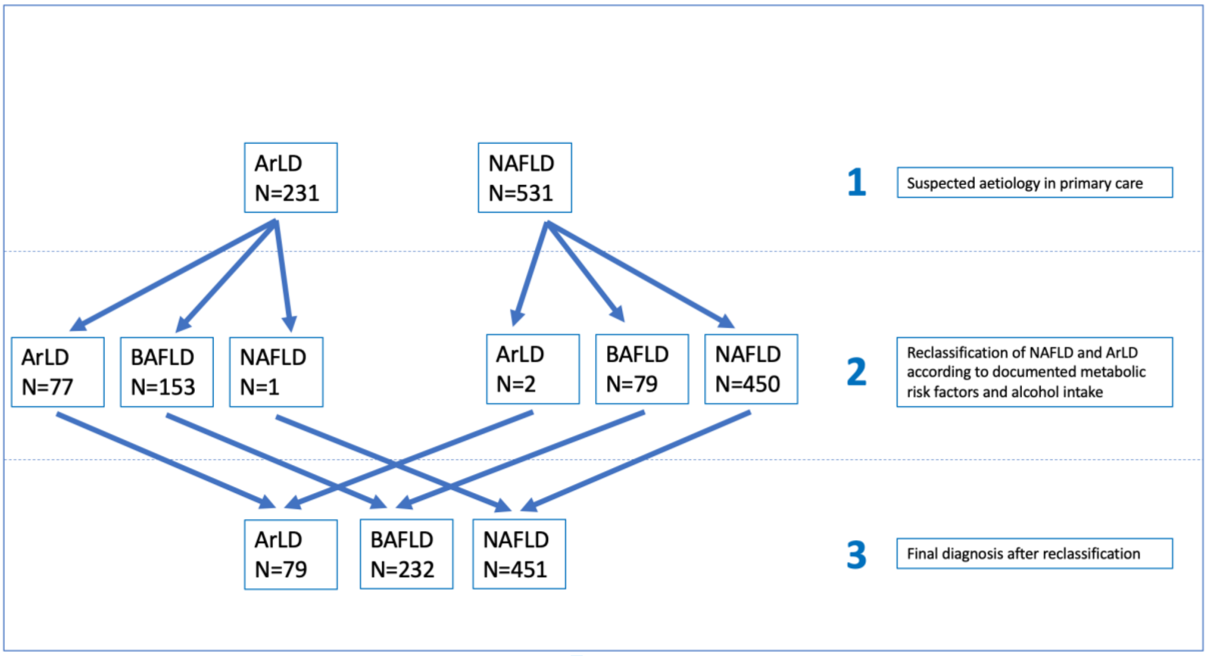

review only

Figure 2: ROC analysis of the performance of indirect tests for fibrosis and simple liver blood tests in the detection of advanced fibrosis (composite clinical judgement) in patients referred with suspected ArLD. (N=231)

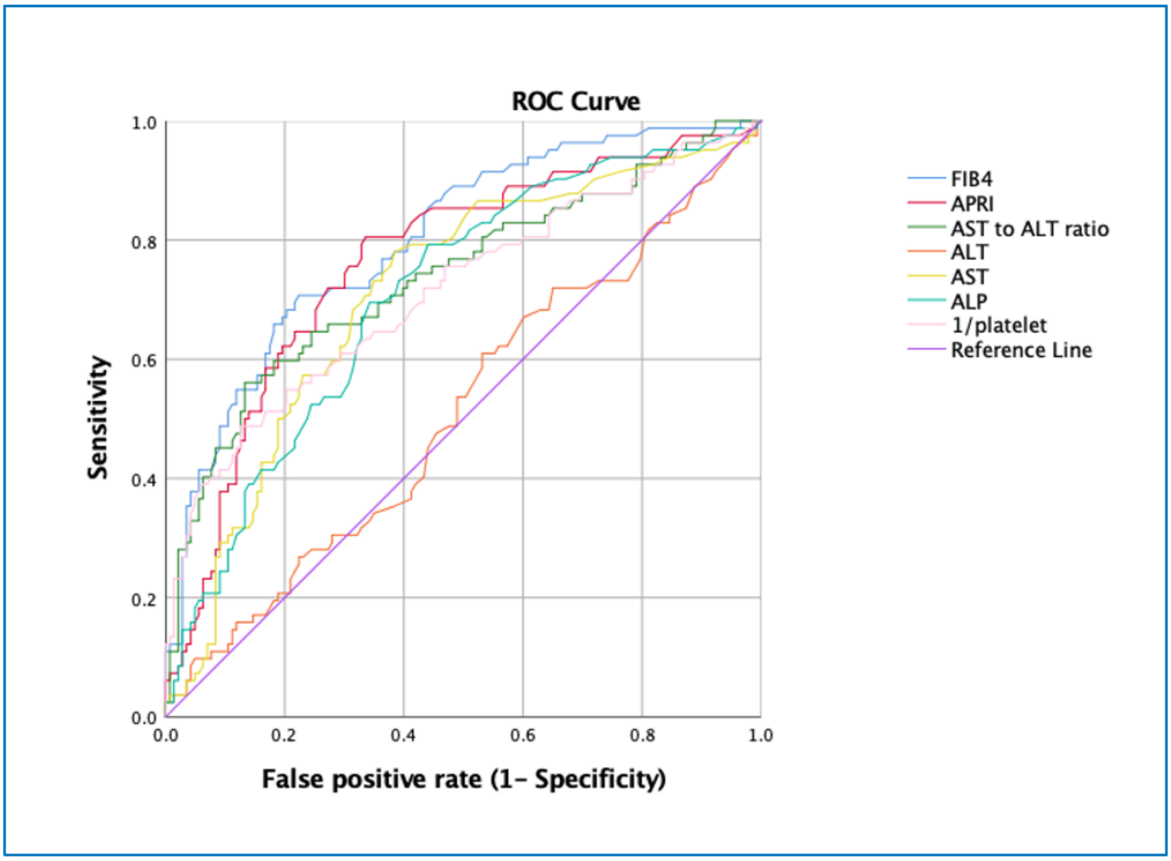

AUROCs with 95% CI in brackets: FIB4: 0.801 (0.742 to 0.860); APRI: 0.763 (0.697 to 0.829); AST:ALT ratio: 0.739 (0.668 to 0.809); ALT: 0.512 (0.433 to 0.591); AST: 0.711 (0.640 to 0.782); ALP: 0.708 (0.638 to 0.777); 1/platelet: 0.714 (0.641 to 0.787). (All p values <0.001 apart from ALT which was non-significant at p = 0.758)

For peer review only

Supplementary table 1: Demographics within each re-classified aetiology group

**Overall characteristics (n=762)**	**ArLD (n =79)**	**NAFLD (n=451)**	**BAFLD (n=232)**	
**Non advanced fibrosis** n (%) **(<F3)**	46/78 (60)	377/450 (83.8)	165/230 (71.7)	*p<0.001*
**Advanced fibrosis** n (%) **(F3 or above)**	32/78 (40)	73/450 (16.2)	65/230 (28.3)	
**Age** (mean; sd)	51.85±13.1	55.3±14.07	57.2±12.3	*p= 0.009*
**BMI** (mean; sd)	21.9± 2.32	32.1±6.17	30.6 ±5.03	*p<0.001*
> 25 n (%)	0/59 (0)	393/443 (88.7)	215/230 (93.3)	*p<0.001*
> 30 n (%)	0/57 (0)	252/416 (60.6)	98/202 (48.5)	*p<0.001*
**Alcohol intake** median U/w (IQR)	79.9 (49.3-140)	0 (0-4)	49.5 (30-88.5)	*p<0.001*
N=	76	434	228	
**Years of harmful drinking**				
Median (IQR)	13 (5-20)	0 (0-0)	20 (8-30)	*p<0.001*
N=	47	427	124	
**ALT** median, (IQR)	43 (28-68)	45 (31-68.25) n	47 (30-67)	*p=0.752*
N=	79	450	232	
**Community ELF score**				
(mean, sd)	9.96 ±0.42	10.33±0.74	10.5±0.84	*p= 0.215*
N=	7	169	54	
**Community FIB4**				*p =0.043*
median	2.75	1.56	2.2	
(IQR)	(1.22-5.19)	(1.38 -2.2)	(1.5-3.25)	
N=	4	75	24	

ArLD = Alcohol-related Liver Disease, NAFLD = Non-Alcoholic-Fatty-Liver-Disease, BAFLD = Both Alcohol and Fatty Liver Disease, sd = standard deviation, BMI = Body Mass Index, IQR = interquartile range, ALT = alanine aminotransferase, ELF = Enhanced Liver Fibrosis score,

For peer review only

STROBE Statement—Checklist of items that should be included in reports of ***cross-sectional studies***

	Item No	Recommendation	Page No
**Title and abstract**	1	(*a*) Indicate the study's design with a commonly used term in the title or the abstract	1
		(*b*) Provide in the abstract an informative and balanced summary of what was done and what was found	4
**Introduction**			
Background/rationale	2	Explain the scientific background and rationale for the investigation being reported	6
Objectives	3	State specific objectives, including any prespecified hypotheses	7
**Methods**			
Study design	4	Present key elements of study design early in the paper	7
Setting	5	Describe the setting, locations, and relevant dates, including periods of recruitment, exposure, follow-up, and data collection	7
Participants	6	(*a*) Give the eligibility criteria, and the sources and methods of selection of participants	7
Variables	7	Clearly define all outcomes, exposures, predictors, potential confounders, and effect modifiers. Give diagnostic criteria, if applicable	7
Data sources/ measurement	8*	For each variable of interest, give sources of data and details of methods of assessment (measurement). Describe comparability of assessment methods if there is more than one group	7
Bias	9	Describe any efforts to address potential sources of bias	7
Study size	10	Explain how the study size was arrived at	7
Quantitative variables	11	Explain how quantitative variables were handled in the analyses. If applicable, describe which groupings were chosen and why	8
Statistical methods	12	(*a*) Describe all statistical methods, including those used to control for confounding	8
		(*b*) Describe any methods used to examine subgroups and interactions	8
		(*c*) Explain how missing data were addressed	n/a
		(*d*) If applicable, describe analytical methods taking account of sampling strategy	n/a
		(*e*) Describe any sensitivity analyses	9
**Results**			
Participants	13*	(a) Report numbers of individuals at each stage of study—eg numbers potentially eligible, examined for eligibility, confirmed eligible, included in the study, completing follow-up, and analysed	10
		(b) Give reasons for non-participation at each stage	n/a
		(c) Consider use of a flow diagram	Fig1
Descriptive data	14*	(a) Give characteristics of study participants (eg demographic, clinical, social) and information on exposures and potential confounders	10
		(b) Indicate number of participants with missing data for each variable of interest	11
Outcome data	15*	Report numbers of outcome events or summary measures	12
Main results	16	(*a*) Give unadjusted estimates and, if applicable, confounder-adjusted estimates and their precision (eg, 95% confidence interval). Make clear which confounders were adjusted for and why they were included	12

For peer review only

For peer review only - http://bmjopen.bmj.com/site/about/guidelines.xhtml

		(*b*) Report category boundaries when continuous variables were categorized	13
		(*c*) If relevant, consider translating estimates of relative risk into absolute risk for a meaningful time period	n/a
Other analyses	17	Report other analyses done—eg analyses of subgroups and interactions, and sensitivity analyses	14
**Discussion**			
Key results	18	Summarise key results with reference to study objectives	15
Limitations	19	Discuss limitations of the study, taking into account sources of potential bias or imprecision. Discuss both direction and magnitude of any potential bias	17
Interpretation	20	Give a cautious overall interpretation of results considering objectives, limitations, multiplicity of analyses, results from similar studies, and other relevant evidence	17
Generalisability	21	Discuss the generalisability (external validity) of the study results	17
**Other information**			
Funding	22	Give the source of funding and the role of the funders for the present study and, if applicable, for the original study on which the present article is based	3

*Give information separately for exposed and unexposed groups.

Note: An Explanation and Elaboration article discusses each checklist item and gives methodological background and published examples of transparent reporting. The STROBE checklist is best used in conjunction with this article (freely available on the Web sites of PLoS Medicine at http://www.plosmedicine.org/, Annals of Internal Medicine at http://www.annals.org/, and Epidemiology at http://www.epidem.com/). Information on the STROBE Initiative is available at www.strobe-statement.org.

For peer review only

BMJ Open

Is there scope to improve the selection of patients with alcohol-related liver disease for referral to secondary care? A retrospective analysis of primary care referrals to a UK liver centre, incorporating simple blood tests.

Journal:	*BMJ Open*
Manuscript ID	bmjopen-2020-047786.R2
Article Type:	Original research
Date Submitted by the Author:	30-Mar-2021
Complete List of Authors:	Rhodes, Freya; UCL, Institute for Liver and Digestive Health, Division of Medicine, Royal Free Campus, Rowland Hill Street, NW3 2PF<br>Cococcia, Sara ; University of Pavia, First Department of Internal Medicine<br>Patel, Preya; UCL, Institute for Liver and Digestive Health, Division of Medicine, Royal Free Campus, Rowland Hill Street, NW3 2PF<br>Panovska-Griffiths, Jasmina; UCL, Department of Applied Health Research; UCL, Institute for Global Health<br>Tanwar, Sudeep; Barts Health NHS Trust, Department of Gastroenterology; UCL, Institute for Liver and Digestive Health, Division of Medicine, Royal Free Campus, Rowland Hill Street, NW3 2PF<br>Westbrook, R; UCL, Institute for Liver and Digestive Health, Division of Medicine, Royal Free Campus, Rowland Hill Street, NW3 2PF<br>Rodger, Alison; UCL, Department of Infection and Population Health<br>Rosenberg, William; UCL, Institute for Liver and Digestive Health, Division of Medicine, Royal Free Campus, Rowland Hill Street, NW3 2PF
**Primary Subject Heading**:	Gastroenterology and hepatology
Secondary Subject Heading:	General practice / Family practice, Public health
Keywords:	Hepatology < INTERNAL MEDICINE, Organisation of health services < HEALTH SERVICES ADMINISTRATION & MANAGEMENT, PUBLIC HEALTH

SCHOLARONE™
Manuscripts

I, the Submitting Author has the right to grant and does grant on behalf of all authors of the Work (as defined in the below author licence), an exclusive licence and/or a non-exclusive licence for contributions from authors who are: i) UK Crown employees; ii) where BMJ has agreed a CC-BY licence shall apply, and/or iii) in accordance with the terms applicable for US Federal Government officers or employees acting as part of their official duties; on a worldwide, perpetual, irrevocable, royalty-free basis to BMJ Publishing Group Ltd ("BMJ") its licensees and where the relevant Journal is co-owned by BMJ to the co-owners of the Journal, to publish the Work in this journal and any other BMJ products and to exploit all rights, as set out in our licence.

The Submitting Author accepts and understands that any supply made under these terms is made by BMJ to the Submitting Author unless you are acting as an employee on behalf of your employer or a postgraduate student of an affiliated institution which is paying any applicable article publishing charge ("APC") for Open Access articles. Where the Submitting Author wishes to make the Work available on an Open Access basis (and intends to pay the relevant APC), the terms of reuse of such Open Access shall be governed by a Creative Commons licence – details of these licences and which Creative Commons licence will apply to this Work are set out in our licence referred to above.

Other than as permitted in any relevant BMJ Author's Self Archiving Policies, I confirm this Work has not been accepted for publication elsewhere, is not being considered for publication elsewhere and does not duplicate material already published. I confirm all authors consent to publication of this Work and authorise the granting of this licence.

For peer review only

Is there scope to improve the selection of patients with alcohol-related liver disease for referral to secondary care? A retrospective analysis of primary care referrals to a UK liver centre, incorporating simple blood tests.

Rhodes F[1*], Cococcia S[1,2*], Patel, P[1], Panovska-Griffiths J[3,4], Tanwar S[5], Westbrook RH[1], Rodger A[4], Rosenberg WM[1]

[1]Institute for Liver and Digestive Health, UCL Division of Medicine, Royal Free Campus, London, UK
[2]First Department of Internal Medicine, San Matteo Hospital Foundation, University of Pavia, Pavia, Italy
[3]Department of Applied Health Research, University College London, UK
[4]Institute for Global Health, University College London, UK
[5] Bart's Health NHS Trust, London, United Kingdom

*joint first author

Author details:

Dr Freya Rhodes (Hepatology research fellow, Institute for Liver and Digestive health, UCL division of medicine, Royal Free Campus, London, UK NW3 2QG)
F.rhodes@doctors.net.uk

Dr Sara Cococcia (Gastroenterology registrar, Institute for Liver and Digestive health, UCL division of medicine, Royal Free Campus, London, UK NW3 2QG and First Department of Internal Medicine, San Matteo Hospital Foundation, University of Pavia, Pavia, Italy)

Dr Preya Patel (Gastroenterology registrar, Institute for Liver and Digestive health, UCL division of medicine, Royal Free Campus, London, UK NW3 2QG)

Dr Jasmina Panovska-Griffiths (Senior research associate in applied statistics, Department of Applied Health Research, University College London, UK and Institute for Global Health, University College London, UK)

Dr Sudeep Tanwar (Consultant Gastroenterologist & Hepatologist, Barts Health NHS Trust, and Honorary Associate Professor, UCL Institute for Liver & Digestive Health)

Dr Rachel Westbrook (Consultant hepatologist, Institute for Liver and Digestive health, UCL division of medicine, Royal Free Campus, London, UK NW3 2QG)

For peer review only

Prof Alison Rodger, Professor of infectious diseases, Institute for Global Health, University College London, UK

Prof William Rosenberg, Professor of Hepatology, Institute for Liver and Digestive health, UCL division of medicine, Royal Free Campus, London, UK NW3 2QG

Address for correspondence: w.rosenberg@ucl.ac.uk

Keywords: Liver fibrosis, Alcohol, NAFLD, referral pathway, non-invasive tests

Abbreviations:

ArLD: Alcohol related Liver Disease
CLD: Chronic Liver Disease
NAFLD: Non-Alcoholic Fatty Liver Disease
BAFLD: Both Alcohol and Fatty Liver Disease
AUD: Alcohol Use Disorder
NIT: Non-Invasive Test
ELF: Enhanced Liver Fibrosis
ALT: Alanine Aminotransferase
AST: Aspartate Aminotransferase
ALP: Alkaline Phosphatase
FIB4: Fibrosis 4 score
APRI: AST to Platelet Ratio Index
BMI: Body Mass Index
T2DM: Type II Diabetes Mellitus
OR: Odds Ratio
CI: Confidence Interval
GPs: General Practitioners
U/w: Units per week

Word count: 4,191, 2 figures, 2 tables

Author contributions: FR performed the analysis of the data, contributed to the collection of the data and wrote the first draft of the manuscript; SC contributed to the collection of data and to the writing of the manuscript; PP contributed to the review and editing of the manuscript, J P-G contributed to the statistical analysis. RW, ST and AR contributed to the analysis

For peer review only

methodology, and the review and editing of the manuscript. WR conceived the study, oversaw the study and reviewed and edited the manuscript drafts. All authors approved the final version of the paper. The corresponding author attests that all listed authors meet authorship criteria and that no others meeting the criteria have been omitted.

Funding

This study is being supported by funding from WMR's National Institute for Health Research Senior Investigator Award (Award number 200249). WMR is an NIHR Senior Investigator and is supported by the NIHR University College London Hospitals Biomedical Research Centre. JPG was supported by the United Kingdom National Institute for Health Research (NIHR) Applied Research Collaboration North Thames (ARC North Thames) at Bart's Health NHS Trust.

Competing interests statement:

WMR is an inventor of the ELF test but receives no related royalties. WMR has received speakers' fees from Siemens Healthineers. The other authors declare no competing interests.

Data sharing statement:

On publication of this article, the dataset will be made available from the corresponding author on reasonable request.

Transparency statement:

The lead author affirms that this manuscript is an honest, accurate and transparent account of the study being reported, and that no important aspects of the study have been omitted, nor discrepancies from the study originally planned.

Exclusive license statement:

The Corresponding Author has the right to grant on behalf of all authors and does grant on behalf of all authors, a worldwide licence to the Publishers and its licensees in perpetuity, in all forms, formats and media (whether known now or created in the future), to i) publish, reproduce, distribute, display and store the Contribution, ii) translate the Contribution into other languages, create adaptations, reprints, include within collections and create summaries, extracts and/or, abstracts of the Contribution, iii) create any other derivative work(s) based on the Contribution, iv) to exploit all subsidiary rights in the Contribution, v) the inclusion of electronic links from the Contribution to third party material where-ever it may be located; and, vi) licence any third party to do any or all of the above."

For peer review only

Abstract:

Objectives

Twenty-percent of people with alcohol-use-disorders develop advanced fibrosis and warrant referral to secondary-care. Improving outcomes in Alcohol-related-Liver-Disease (ArLD) relies on its earlier detection in primary-care with non-invasive-tests (NIT). We aimed to determine the proportion of alcohol-related referrals who were diagnosed with advanced fibrosis in secondary-care, the prevalence of 'BAFLD' (Both Alcohol and Fatty Liver Disease), and the potential impact of NIT on referral-stratification.

Design/setting

Retrospective analysis of all GP-referrals with suspected ArLD/NAFLD to a UK hepatology-centre between Jan2015-Jan2018.

Participants

Of 2,944 new referrals, 762 (mean age 55.5±13.53 years) met inclusion-criteria: 531 NAFLD and 231 ArLD, of which 147 (64%) could be reclassified as 'BAFLD'.

Primary outcome-measure: Proportion of referrals with suspected ArLD/NAFLD with advanced fibrosis as assessed by tertiary-centre hepatologists using combinations of FibroScan, imaging, examination and blood tests, and liver histology where indicated.

Secondary outcome-measures: Included impact of BMI/alcohol consumption on the odds of a diagnosis of advanced fibrosis, and performance of NIT in predicting advanced fibrosis in planned post-hoc analysis of referrals.

Results:

Amongst ArLD referrals 147/229 (64.2%) had no evidence of advanced fibrosis and were judged 'unnecessary'. Advanced fibrosis was observed in men drinking ≥50U/w (OR 2.74, 95% CI 1.51-to-5.00, p = 0.001), and ≥35U/w in women (OR 5.11, 95% CI 1.31-to-20.03, p = 0.019). Drinking > 14 U/w doubled the likelihood of advanced fibrosis in overweight/obesity (OR 2.11; CI 1.44-to-3.09; p<0.001). Use of FIB4 could halve unnecessary referrals (OR 0.50; CI 0.32-to-0.79, p = 0.003) with false-negative rate of 22%, but was rarely used.

Conclusions:

The majority of referrals with suspected ArLD were deemed unnecessary. NIT could improve identification of liver damage in ArLD, BAFLD and NAFLD in primary-care. Anecdotal thresholds for harmful-drinking (35U/w in women and 50U/w in men) were validated. The impact of alcohol on NAFLD highlights the importance of multi-causality in CLD.

For peer review only - http://bmjopen.bmj.com/site/about/guidelines.xhtml

Article Summary

Strengths and limitations of this study

- This study reflects real-world experience of consecutive alcohol referrals from primary care to a specialist liver centre over a 3-year period.
- Results of tests routinely performed in primary care can be used to improve selection of patients for referral.
- This was a retrospective study relying on data held in electronic clinical records, including of self-reported alcohol intake.
- Our study used consensus judgement of expert hepatologists to assess liver disease rather than liver biopsy as a reference standard to assess fibrosis severity.

For peer review only

Introduction

Approximately 90% of all chronic liver disease (CLD) is preventable, with the commonest causes of cirrhosis attributed to ArLD and NAFLD (1). Mortality from cirrhosis has increased 400% since 1970, predominantly due to alcohol, although the rising prevalence of NAFLD is contributary (2). Hepatic steatosis develops in up to 90% of people with Alcohol Use Disorder (AUD) or obesity (3, 4), but advanced fibrosis or cirrhosis will affect only approximately 20% of people with AUD (5) and 5% with NAFLD (6). Both AUD and obesity can be managed effectively in primary-care but advanced fibrosis and cirrhosis warrant management by liver specialists in secondary care. Detecting the minority of patients requiring specialist care is challenging because advanced fibrosis and most cases of cirrhosis are asymptomatic and simple liver blood tests (LFTs) and ultrasound imaging are neither sensitive nor specific in detecting advanced fibrosis or cirrhosis (7). As a consequence, three-quarters of people with CLD first present to healthcare with established advanced liver disease when behaviour change or therapeutic interventions have only modest impacts on prognosis (1, 8, 9).

Conversely, as many as 92% of people referred to secondary-care with suspected CLD do not have advanced fibrosis or cirrhosis requiring specialist care and could have remained in primary-care for ongoing management (10). Pathways of care employing the use of NITs for liver fibrosis (FIB-4) and the Enhanced Liver Fibrosis Test (ELF) in primary-care have been shown to be effective in the management of NAFLD, yielding an 88% reduction in 'unnecessary referrals' to liver specialists with a five-fold increase in the detection of advanced fibrosis and cirrhosis, and significant cost-savings (10, 11), influencing national guidelines(12). However, the proportion of referrals with AUD who do not have advanced ArLD that could be considered 'unnecessary' is unknown.

The ELF test has also been used successfully to triage patients from primary to secondary-care with AUD in Denmark (13). While current UK national guidelines recommend consideration of NIT in people with AUD in primary care (7), alcohol pathways employing NIT are not widely established in the UK and none have been evaluated to our knowledge.

Although NAFLD and ArLD are described as distinct entities for research purposes, the risk factors for both conditions co-exist in many patients. Moreover, it is increasingly recognised that alcohol and fat interact to cause liver damage, with obese people having increased risks of liver fibrosis for any given alcohol intake (7, 14-18). In this study we aimed to determine the proportion of patients referred for investigation of ArLD from primary-care to secondary-

For peer review only - http://bmjopen.bmj.com/site/about/guidelines.xhtml

care hepatology clinics that had evidence of advanced fibrosis; and the prevalence of both alcohol and fat as co-contributing factors to CLD, termed 'BAFLD' to describe the combination of Both Alcohol and Fatty Liver Disease (19). In addition, we aimed to determine the performance of simple NITs in the identification of cases of advanced fibrosis.

Methods

Study design

This is a retrospective cross-sectional analysis of consecutive patients aged ≥18 years newly referred from primary-care to a hospital-based hepatology service at the Royal Free London NHS Foundation Trust (RFL), with a suspected diagnosis of ArLD or NAFLD between January 2015 and January 2018. Patients were excluded if they had any other hepatological diagnosis made prior to referral (supplementary table 1).

Outcome measures

The primary outcome measure was the proportion of new patients referred from GP to hepatology clinic with suspected ArLD that had advanced fibrosis and could be deemed 'necessary' referrals.

Secondary outcome measures included the prevalence of 'BAFLD' amongst patients referred with suspected ArLD or NAFLD, analysis of demographic data as potential risk factors for a diagnosis of advanced fibrosis (including BMI, alcohol consumption, smoking status, age, sex, and deprivation score), and a post-hoc analysis of the performance of FIB4 and APRI in predicting a diagnosis of advanced fibrosis.

Study population

All electronic GP referrals for suspected ArLD or NAFLD during this period were reviewed in order to identify cases referred for NAFLD who were subsequently found to be drinking hazardous amounts of alcohol (>14 units per week). As these conditions were not always reliably coded and triaged from the outset, every new referral from GP to hepatology clinic during this time period was reviewed in order to select out the NAFLD and ArLD referrals to ensure cases were not missed. Sample size was based upon 3-years' worth of referrals.

'Suspected ArLD' referrals were defined as those in which the GP referral letter requested an assessment by a liver specialist specifying concerns about suspected ArLD or expressing concerns about a patient's alcohol intake.

'Suspected NAFLD' referrals were defined as those in which the GP referral letter either specified that they were referring the patient to hepatology 'with suspected NAFLD' or 'on

For peer review only - http://bmjopen.bmj.com/site/about/guidelines.xhtml

the local NAFLD referral pathway', OR, in the absence of any other cause of liver dysfunction, where the GP specified that the patient had steatosis or chronic liver disease on ultrasound in combination with mentioning metabolic risk factors (BMI ≥25, diabetes, high waist circumference, high cholesterol or hypertension).

Data Collection

Anonymised data were extracted from the patients' electronic records. These included demographics, reason for referral, deprivation score, weight, height, waist circumference, alcohol intake, comorbidities, and any fibrosis assessment before and after referral. Where weight and height were unavailable, but clinical records reported that the patient was overweight or obese, they were categorised accordingly to BMI >25 (overweight) or BMI >30 (obese). FIB4 and APRI scores were calculated using the blood tests from the first attendance to clinic after referral.

The diagnosis of advanced fibrosis (equivalent to a histological stage of ≥ F3/4) or cirrhosis (≥ F4) was established by expert clinical judgement by hepatologists based on a composite of FibroScan, imaging, blood tests, clinical examination and liver histology where available, and this information was extracted from the electronic medical records. In the minority of cases where a diagnosis of advanced fibrosis was not clearly documented, decisions were reviewed by the study team (FR and SC) and consensus achieved. FibroScan was considered diagnostic for advanced fibrosis if the elasticity of a valid scan was ≥11kpa in ArLD (12, 20) and ≥10kpa in NAFLD patients (21). For variables where any data were missing, the denominator used in the analysis was adjusted for only available data.

'Unnecessary referrals' were defined as those patients that, subsequent to an assessment by a liver specialist, were deemed not to have advanced fibrosis and could be discharged back to ongoing care in the community.

In light of the frequent overlap between the two conditions, patients were subsequently recoded as having Both Alcohol and Fatty Liver Disease (BAFLD) if ArLD and NAFLD risk factors were both present. More specifically, BAFLD was applied to patients referred for suspected NAFLD who were subsequently found to be drinking more than 14 units of alcohol per week; and to patients who were referred for suspected ArLD, who also had either a BMI >25, or features of the metabolic syndrome. The metabolic syndrome was defined according to the International Diabetes Federation (IDF) and American Heart Association (AHA) as the presence of at least three of the following criteria: enlarged waist circumference (≥94cm in

For peer review only - http://bmjopen.bmj.com/site/about/guidelines.xhtml

European men, ≥90cm South Asian men, ≥80cm women), hypercholesterolaemia, hypertension and type 2 diabetes (22).

Statistical analysis

Descriptive statistical analyses included calculations of the frequencies and percentages for categorical variables, while for continuous data means and standard deviation (SD) for normally distributed data, or medians and interquartile range (IQR) for skewed data were used. For the comparison of categorical variables, Chi-Squared or Fischer's exact test was used (the latter when n = <5), and for continuous data Mann Whitney-U or Student's-t test depending on the data distribution.

For data with more than three variables to compare, ANOVA or Kruskall Wallis ANOVA were used, depending on the distribution of the data.

Alcohol consumption was categorised into groups of units per week according to the perceived risk of liver damage established in the literature (7) (0-35, 36-50,51-100, >100 units per week) and into quartiles of the population distribution of alcohol consumption for the ArLD cohort in which few patients were drinking <50 units per week. Multiple binary logistic regression analysis was used to determine the association between key variables and the presence of advanced fibrosis. The key variables were those risk factors for fibrosis that were of established importance in the literature, and those associated with p values <0.25 in the univariate analysis. All p values were 2-sided and significance set at <0.05. All data were analysed using SPSS software (Version 25.0. Armonk, NY: IBM Corp), except for the odds ratios (ORs) for differences in outcomes for modelling of data with FIB4 compared with current practice, together with 95% confidence intervals and chi-square for statistical significance which were performed using MedCalc statistical software 2018.

Ethics

This study uses secondary anonymised patient data. The project was registered with the Integrated Research Application System (IRAS 272448) and judged to not require ethical approval or informed consent according to Health Research Authority guidance as it comprises data that were collected routinely as part of a registered service evaluation at the Royal Free London NHS Foundation Trust.

For peer review only

Patient and Public involvement

Patients and the Public were not involved in this study.

RESULTS

Patient demographics:

Between January 2015 and January 2018, a total of 2,944 patients were referred to the RFL hepatology service from primary care and of these, 762 (mean age 55.5±13.53 years) met the inclusion criteria for this study; 231 patients were referred with suspected ArLD (mean age 54.68±12.37 years), and 531 with suspected NAFLD (mean age 55.88±14 years). One patient was deemed to have active hepatitis C virus infection as comorbidity and three were found to have inactive chronic hepatitis B after referral. The demographic characteristics of the included patients are reported in **Table 1.** There was a higher proportion of male patients in the ArLD group (76.2%) than amongst the NAFLD group (54.2%, $p<0.001$). Active or previous smoking was significantly more common among those referred for ArLD compared to the NAFLD group (47.1% vs 11.3%; $p<0.001$). The average BMI was significantly higher in the NAFLD group than the ArLD group (31.9 and 27.9 kg/m^2 respectively, $p<0.001$), while median alcohol consumption was significantly higher in the ArLD group at 70 units/week (42-135), compared to 0 units/week (0-7) in the NAFLD group. The majority of the study population lay within the lowest 4 deciles of deprivation, and no significant difference in levels of deprivation was seen when ArLD and NAFLD referrals were compared ($p=0.326$).

For peer review only - http://bmjopen.bmj.com/site/about/guidelines.xhtml

Table 1: Baseline characteristics

Patient characteristics	Overall (n=762)	Suspected ArLD referrals* (n =231)	Suspected NAFLD referrals** (n=531)	
**Age** (mean; sd)	55.52 ±13.53	54.68±12.37	55.88±14	*p = 0.262*
**Male** n (%)	464 (60.9%)	176 (76.2%)	288 (54.2%)	*p <0.001*
**BMI** (mean; sd)	30.85 ± 6.23	27.9 ± 5.46 (n=174)	31.9 ±6.15	*p <0.001*
> 25 n (%)	608/732 (83.1)	149/211 (70.6)	459/521 (88.1)	*p <0.001*
> 30 n (%)	350/675 (51.9)	56/185 (30.3)	294/490 (60)	*p <0.001*
**Alcohol intake** U/w (median, IQR)	5, (0-42.75)	70 (42-134.8)	0 (0-7)	*p <0.001*
N =	738	226	512	
**Years of harmful drinking**				
Median (IQR)	0 (0-3)	20 (6-30)	0 (0-0)	*p <0.001*
Total n =	598	143	455	
**Diabetes** n (%)	235/760 (30.9)	38/231 (16.5)	197/529 (37.2)	*p <0.001*
**Hypertension** n (%)	397/761 (52.2)	113/231 (48.9)	284/530 (53.6)	*p=0.236*
**Hypercholesterolaemia** n (%)	352/759 (46.4)	81/231 (35.1)	271/528 (51.3)	*p <0.001*
**Smoking status:** Non- smoker n (%)	369/681 (54.2)	65/204 (31.9)	304/477 (63.7)	*p<0.001*
Smoker n (%)	150/681 (22)	96/204 (47.1)	54/477 (11.3)	
Ex- smoker n (%)	162/681 (23.8)	43/204 (21.1)	119/477 (24.9)	
**ALT** median (IQR)	45 (30-67)	47 (30-68)	45 (30-67)	*p =0.360*
N =	761	231	530	
**Deprivation score rank** Median (IQR)	11314 (6451-17642)	10648 (6100-17464)	11637 (6578-17761)	*p =0.326*
**Deprivation score decile**: 1	51 (6.7%)	12 (5.2%)	39 (7.3%)	*p=0.264*
2	146 (25.9%)	53 (28.1%)	93 (24.9%)	
3	134 (43.4%)	42 (46.3%)	92 (42.2%)	
4	107 (57.5%)	30 (59.3%)	77 (56.7%)	
5	101 (70.7%)	33 (73.6%)	68 (69.5%)	
6	82 (81.5%)	26 (84.8%)	56 (80%)	
7	64 (89.9%)	17 (92.2%)	47 (88.9%)	
8	44 (95.7%)	8 (95.7%)	36 (95.7%)	
9	22 (98.6%)	6 (98.3%)	16 (98.7%)	
10	11 (100%)	4 (100%)	7 (100%)	
**Had Biopsy** n (%)	122/762 (16%)	10/231 (4.3%)	112/531 (21.1.%)	*p <0.001*
**Had FibroScan** n (%)	575/762 (75.5%)	158/231(68.4%)	417/531 (78.5%)	*p =0.003*
**Valid FibroScan reading*****	524/575 (91%)	140/158 (89%)	389/417 (93%)	
**FibroScan** median Kpa (IQR)	5.5 (4.5-7.7)	6 (4.7-8.5)	5.4 (4.4-7.5)	*p = 0.03*

* Where primary reason for referral from GP was for suspected alcohol-related liver disease
**Where primary reason for referral from GP was for suspected NAFLD
*** FibroScan results were considered invalid if: IQR/M >30%, success rate <60%, <10 valid readings, or if this information was not recorded in the FibroScan report (missing information about IQR/M ratio/success rate made up n=22/575 FibroScan results).
SD = standard deviation, IQR = interquartile range, ALT = alanine aminotransferase, BMI = body mass index,

Reasons for referral from primary care

The presence of hepatic steatosis on an ultrasound scan and abnormal LFTs were the commonest reasons for referral to hepatology clinic regardless of the aetiology. These were followed by elevated ELF and FIB4 in the NAFLD cohort (38.2 %and 16.9% respectively).

For peer review only

Only 38/231 (16.4%) of patients with suspected ArLD had a NIT in primary-care prior to referral (25 ELF scores, 13 FIB4) and of these, 25/38 (66%) patients had comorbid features of the metabolic syndrome and so were subsequently recoded as BAFLD. Amongst the NAFLD referrals 293/531 (55.2%) had a NIT prior to referral in accordance with the local NAFLD pathway. Of these patients 203/293 (69%) were referred on the basis of an elevated ELF test and 90/293 (31%) based on their FIB4 score.

Prevalence of advanced fibrosis in patients referred with suspected ArLD or NAFLD.

Data on fibrosis stage were available for 758/762 patients following hepatology review, with four not attending for assessment. Of patients with suspected ArLD, 64.2% (147/229) had no evidence of advanced fibrosis and could be discharged back to primary-care. This figure was even higher in the NAFLD cohort with 83.4% not having advanced fibrosis.

Of the patients referred with suspected ArLD who had advanced fibrosis (82/229), the frequency with which fibrosis tests were used were: liver biopsy in 10% (8/82), FibroScan in 41% (34/82) and radiology in 62% (51/82).

Of the patients referred with suspected NAFLD who had advanced fibrosis (88/529), the frequency with which fibrosis tests were used were: liver biopsy in 47% (41/88), FibroScan in 64% (56/88) and radiology in 33% (29/88).

Risk of advanced fibrosis (>/F3) in patients referred with suspected ArLD.

Univariate analysis of the 231 patients referred with ArLD revealed that advanced fibrosis was associated with raised ALP (OR 1.012, 95% CI 1.006 to 1.018 p <0.001) and higher alcohol consumption (alcohol data available for 224/231) (OR 1.006, 95% CI 1.002 to 1.010, p=0.006). When categorised into alcohol unit groups of: <35 U/w, 36-50 U/w, 51-100 U/w, >101 U/w; patients drinking >50 U/w had a higher risk of advanced fibrosis in this cohort (OR 2.899, 95% CI 1.068 to 7.869, p= 0.037). The multivariable logistic regression model found that the odds of advanced fibrosis in suspected ArLD was independently associated with increased units of alcohol consumed, (OR 1.007, 95%CI 1.002-1.012, p=0.007), ALP (OR 1.009, 95% CI 1.002-1.016, p=0.01), and reduced platelets (OR 0.992, 95%CI 0.988-0.996, p<0.001). There was a trend towards higher odds of advanced fibrosis with increased age, but this did not reach significance (p=0.059).

For peer review only - http://bmjopen.bmj.com/site/about/guidelines.xhtml

Patients with risk factors for both ArLD and NAFLD: 'BAFLD'.

Patients with risk factors for both ArLD and NAFLD were classified as BAFLD (as defined earlier) and the whole cohort was re-classified into three categories: ArLD, NAFLD and BAFLD, in order to evaluate further risk factors for advanced fibrosis **(figure 1).**

From the GP referral letters, 147 (63.6%) patients out of the 231 patients referred to the hepatology clinic with suspected ArLD were overweight, or met the diagnostic criteria of the metabolic syndrome and were therefore reclassified as BAFLD. Of the 531 patients referred to hepatology as suspected NAFLD, 80 of them (15.1%) also regularly consumed an average of more than 14 units per week and were reclassified as BAFLD. Overall, 83.1% of the whole cohort were overweight and 50% obese. As expected, the proportion of patients who were overweight and obese was significantly higher in the NAFLD cohort compared to ArLD cohort ($p<0.001$). The main characteristics of the three cohorts can be found in supplementary table 2.

Patients with BAFLD had almost double the prevalence of advanced fibrosis when compared to NAFLD (29% and 16.2% respectively, (OR 2.11, 95% CI 1.441 to 3.094), $p <0.001$, suggesting that hazardous drinking doubled the risk of fibrosis in people who are overweight or obese in this study population.

Patients in the ArLD cohort had the highest prevalence of advanced fibrosis (38%), and their weekly alcohol intake was almost double that of the BAFLD patients, precluding the opportunity to compare the impact of overweight/obesity on heavy alcohol consumption in this cohort.

Influence of alcohol on fibrosis risk

As the number of ArLD patients drinking <50 units per week (U/w) was small, the entire cohort (n=762) was examined in an attempt to identify a potential threshold for the effect of alcohol on fibrosis risk. Other factors influencing fibrosis risk including age and BMI were also studied. Alcohol data were available for 734/762 patients.

Increased alcohol U/w predicted advanced fibrosis (OR 1.009, 95%CI 1.006 to 1.012, $p = <0.001$) on univariate analysis.

Alcohol units were categorised into quartiles of the reported distribution of consumption (0-42 U/w, 43-70 U/w, 71-135 U/w, >136 U/w). Binary logistic regression revealed that patients consuming ≥43 U/w were at greater risk of advanced fibrosis than those drinking less than 43 U/w. (OR 1.814, 95%CI 1.038 to 3.172, $p = 0.037$), and those drinking ≥70 U/w were at

For peer review only - http://bmjopen.bmj.com/site/about/guidelines.xhtml

more than four times the risk of having advanced fibrosis compared with those drinking less than 43 U/w (OR 4.25, 95% CI 2.334 to 7.740, p = <0.001).

Alcohol consumption was then evaluated at literature-based unit thresholds of interest (0-35 U/w, 36-50 U/w, 51-100 U/w, >101 U/w) revealing that drinking more than 35 U/w was associated with double the odds of developing advanced fibrosis compared with those drinking <35 U/w (OR 2.173, 95% CI 1.119 to 4.219, p = 0.022) and the odds increased to over five-fold in those drinking more than 100 units per week (OR 5.044, 95% CI 3.071 to 8.284, p <0.001).

A different threshold effect was found when these data were analysed separately for men and women. In the overall cohort of 762 patients, the risk of having advanced fibrosis was higher in those men drinking >50 U/w (OR 2.743, 95% CI 1.506 to 4.998, p = 0.001), while in women the risk of having advanced fibrosis increased significantly at only >35 U/w (OR 5.115, 95% CI 1.306 to 20.030, p = 0.019), compared to <35 U/w).

In the overall cohort of 762 patients with ArLD/NAFLD/BAFLD (of which complete data for this model were available for 625/762), multivariable regression analysis revealed that increased units of alcohol, age, ALP, BMI and decreased platelet count were significantly associated with increased odds of a diagnosis of advanced fibrosis.

Modelling the impact of indirect fibrosis tests on the detection of advanced fibrosis in patients referred from primary care with suspected ArLD.

Blood test results from the first attendance at the secondary care were used to calculate FIB4 and APRI scores for 225/231 patients referred with suspected ArLD (6 patients did not have an AST value available). Median FIB4 and APRI were 1.58 (IQR 0.97-3.29) and 0.68 (IQR 0.36-1.53) respectively.

Both scores independently predicted the clinical diagnosis of advanced fibrosis in secondary-care in multivariable regression analysis (for FIB4, OR=1.658, 95% CI 1.397 to 1.967, p <0.001; for APRI, OR=1.485, 95% CI 1.204 to 1.832, p <0.001).

When ROC analysis was used to examine the ability of NIT based on routine blood tests to predict a diagnosis of advanced fibrosis, FIB4 performed the best (AUROC 0.801), compared with APRI, AST, ALT, ALP and platelet count (All p <0.005 using DeLong comparison) and numerically but not significantly better than APRI (p = 0.06) **(figure 2).**

Amongst the cohort of patients with ArLD referred to secondary-care, 35.81% were judged to have advanced fibrosis and thus 64.2% could be considered 'unnecessary' referrals. Use of a FIB4 threshold of ≥3.25 (23) could have improved the detection of patients with advanced

For peer review only - http://bmjopen.bmj.com/site/about/guidelines.xhtml

fibrosis nearly five-fold (OR=*4.82; 95% CI 2.56 to 9.09, p <0.0001)*, leading to a 79.3% reduction in unnecessary referrals to secondary care (64.2% to 27.1%) (OR = 0.21; 95% CI 0.11 to 0.39, p <0.001) However, this would be associated with the exclusion of 39 patients judged to have advanced fibrosis (false negative rate of 47.6%). **(Table 2).**

When modelling the referrals using a FIB4 threshold of ≥1.45,(23) the detection of advanced fibrosis improved two-fold compared with standard-care (OR=1.98; 95% CI 1.27 to 3.09, p = 0.0027) and reduced the number of unnecessary referrals from 64.2% to 47.5% (OR=0.5; CI 0.32 to 0.79, p = 0.003), with 103 patients (45.7%) having a FIB4 score below 1.45 that could have remained in primary care. The false negative rate was lower using FIB4 ≥1.45compared to threshold ≥3.25 (18/103, 22% compared to 39/103, 47.5%; X^2=10.60; p=0.001).

Table 2: Accuracy of indirect fibrosis markers in detecting advanced fibrosis in a cohort of 231 patients referred from primary care with suspected ArLD. (N= 225/231.)

Indirect fibrosis test (n=225/231)	Correctly classifies	Sensitivity (95% CI)	Specificity (95% CI)	PPV (95% CI)	NPV (95% CI)	LR+ (95% CI)	LR- (95% CI)	TP FP	FN TN	False negative rate (%)	False Positive rate (%)
**APRI ≥ 1**	165 (73.3%)	64.6% (54-75)	78.3% (70-85)	63.1% (52-73)	80% (72-86)	3.02 (2.13-4.28)	0.44 (0.33-0.6)	53 31	29 112	35.4	21.7
**FIB4 ≥ 3.25**	170 (75.6%)	52.4% (41.2-63.5)	88.8% (82.2-93.3)	72.9% (59.5-83.3)	76.5% (69.1-82.6)	4.69 (2.83-7.77)	0.54 (0.43-0.67)	43 16	39 127	47.6	11.2
**FIB4 ≥1.45**	149 (66%)	78% (67.3-86.1)	59.4% (50.9-67.4)	52.4% (43.3-61.5)	82.5% (73.5-89)	1.92 (1.53-2.42)	0.37 (0.24-0.56)	64 58	18 85	22	40.6

For peer review only

Discussion

Two-thirds of the patients referred to secondary-care for suspected ArLD had no evidence of advanced fibrosis, representing unnecessary referrals. This can be explained in part because the commonest reasons for referral were abnormal LFTs and ultrasound scans, neither of which are sensitive or specific tests for advanced fibrosis (7). While some of these patients may have benefited from a hepatologist's advice about the wider consequences of their drinking, many primary-care physicians consider that they are better placed to deliver brief advice about hazardous or harmful drinking and referral to liver specialists should be

restricted to patients with ArLD. Only 38/231 patients with suspected ArLD had any kind of fibrosis assessment prior to referral to secondary-care, the majority of whom had features of metabolic syndrome or were overweight and received FIB4 and ELF tests suggesting that their GPs had followed the local NAFLD pathway that incorporates these investigations. These patients were reclassified as having BAFLD.

The majority (64%) of patients referred with suspected ArLD were overweight, obese or had features of metabolic syndrome. These patients with BAFLD had double the odds of advanced fibrosis when compared to the NAFLD cohort suggesting that hazardous drinking is associated with a doubling of the risk of liver fibrosis in people who are overweight or obese. This both highlights the increased risk of liver disease in patients with dual pathology and the importance of considering multimorbidity in chronic liver disease.

Although national guidelines state that the risk of advanced fibrosis develops at a lower alcohol unit threshold for women than men (<35 U/w for women, <50 U/w for men), (7) these thresholds are not based on published data that we have been able to identify. Few studies have investigated the association between levels of alcohol consumption and the risk of advanced fibrosis, and those that did have reported a range of thresholds (24-29). Furthermore, the levels of drinking that cause harm in the context of overweight and obesity are not known but we derived these same thresholds of 35 U/w in women and 50 U/w in this cohort of 762 patients that included a high prevalence of overweight and obese people. It should be noted that these thresholds focus purely on the risk of advanced liver fibrosis and cannot be generalized to other health measures. National guidelines state that there is an increased risk to health above 14 U/w.

The performance of "indirect" serum fibrosis tests is well reported in NAFLD, but less so in ArLD. In this study cohort of 231 ArLD patients, FIB4 and APRI outperformed simple liver blood tests (ALP, ALT, AST and platelet count) in predicting a diagnosis of advanced fibrosis on AUROC analysis, with FIB4 having the highest AUROC of 0.801. However, when examining FIB4 at literature-derived binary thresholds of 3.25 and 1.45, (13, 23) it did not perform as well in detecting clinically defined advanced fibrosis as has been reported in a recent study in which all participants were required to undergo liver biopsy (13). Stratifying patients in primary care using a FIB4 threshold of 3.25 could have reduced unnecessary referrals by 79.3%, with PPV and NPV for the detection of advanced fibrosis of 72.9% and 76.5% respectively. However, the associated false negative rate was 47.5% suggesting that nearly half the cases of advanced fibrosis would be left in primary care, making it unsuitable for case stratification. A FIB4 threshold of 1.45 produced a lesser, but still significant, false negative

For peer review only - http://bmjopen.bmj.com/site/about/guidelines.xhtml

rate of 22%, and although it reduced the proportion of unnecessary referrals by 50%, the PPV was 52.4% and overall, this threshold correctly classified only 66% of patients into presence or absence of advanced fibrosis. These results suggest that an effective ArLD pathway would require the use of either a NIT with better diagnostic performance or the use of two or more NIT in series, as employed in the Camden and Islington NAFLD pathway (10) .

This retrospective study lacked access to liver biopsy as a reference-standard to stage fibrosis severity. Self-reported alcohol intake at the point of referral to secondary-care was used to record drinking behavior and this may not be reliable. However, this clinic-based sample of 'real-world' cases reflects current practice in the UK and many other countries and highlights the opportunity to stratify patients with ArLD community settings to ensure that only those with a high likelihood of advanced fibrosis are referred for liver specialist care.

Having so many 'unnecessary referrals' to secondary-care is not only an inefficient use of resources, but also exposes patients to unnecessary investigation and the associated time, risk and anxiety. These patients could be managed more appropriately in community settings with an appropriate focus on the wider harms associated with their drinking. Conversely emphasis on those with advanced fibrosis might improve the early detection of those drinkers who are likely to progress to cirrhosis and suffer life-limiting effects of their drinking.

Based on the performance of APRI and FIB4 in this cohort, we would not recommend their routine use to risk stratify patients with AUD. Instead, further evaluation of pathways incorporating non-invasive tests such as ELF or FibroScan (7, 12) (13) would be preferable.

This study highlights the multi-causality and multi-morbidity endured by patients with ArLD and NAFLD. Although the interaction between alcohol and obesity is recognized, the low threshold of alcohol consumption at which the risk of advanced fibrosis nearly doubled in this cohort highlights the importance of communicating this risk to patients with fatty liver disease in clinics and through public health messaging. There is a need for greater awareness amongst healthcare professionals, policy makers and the public and a need for a multi-disciplinary approach to address the lifestyle risk factors that are likely to influence the morbidity and mortality of those with BAFLD.

In summary, the current referral strategy for patients with alcohol use disorders at risk of liver disease from primary care is inefficient and ineffective. There is a need for increased awareness of the need to search for fibrosis using appropriate strategies incorporating non-invasive testing, and education of the guidelines for fibrosis testing in both AUD and NAFLD. In addition, there is a need for improved collaboration between primary and secondary-care services to develop referral pathways employing NIT, with evaluation to further refine

For peer review only - http://bmjopen.bmj.com/site/about/guidelines.xhtml

thresholds for referral and education to improve awareness and the advice provided to patient about the impact of overweight/obesity and alcohol on liver health.

Figure Captions

Figure 1: Flow chart depicting reclassification of aetiologies

Figure 2: ROC analysis of the performance of indirect tests for fibrosis and simple liver blood tests in the detection of advanced fibrosis (composite clinical judgement) in patients referred with suspected ArLD. (N=231)

AUROCs with 95% CI in brackets: FIB4: 0.801 (0.742 to 0.860); APRI: 0.763 (0.697 to 0.829); AST:ALT ratio: 0.739 (0.668 to 0.809); ALT: 0.512 (0.433 to 0.591); AST: 0.711 (0.640 to 0.782); ALP: 0.708 (0.638 to 0.777); 1/platelet: 0.714 (0.641 to 0.787). (All p values <0.001 apart from ALT which was non-significant at p = 0.758)

For peer review only

References

1. British-Liver-Trust. Alcohol Related Liver Disease: Statistics 2019 [Available from: https://britishlivertrust.org.uk/information-and-support/living-with-a-liver-condition/liver-conditions/alcohol/.]
2. Williams R, Aspinall R, Bellis M, Camps-Walsh G, Cramp M, Dhawan A, et al. Addressing liver disease in the UK: a blueprint for attaining excellence in health care and reducing premature mortality from lifestyle issues of excess consumption of alcohol, obesity, and viral hepatitis. The Lancet. 2014;384(9958):1953-97.
3. Younossi ZM, Koenig AB, Abdelatif D, Fazel Y, Henry L, Wymer M. Global epidemiology of nonalcoholic fatty liver disease-Meta-analytic assessment of prevalence, incidence, and outcomes. Hepatology. 2016;64(1):73-84.
4. Gao B, Bataller R. Alcoholic liver disease: pathogenesis and new therapeutic targets. Gastroenterology. 2011;141(5):1572-85.
5. Teli MR, Day CP, Burt AD, Bennett MK, James OF. Determinants of progression to cirrhosis or fibrosis in pure alcoholic fatty liver. Lancet. 1995;346(8981):987-90.
6. Vernon G, Baranova A, Younossi ZM. Systematic review: the epidemiology and natural history of non-alcoholic fatty liver disease and non-alcoholic steatohepatitis in adults. Aliment Pharmacol Ther. 2011;34(3):274-85.
7. Newsome PN, Cramb R, Davison SM, Dillon JF, Foulerton M, Godfrey EM, et al. Guidelines on the management of abnormal liver blood tests. Gut. 2018;67(1):6-19.
8. Sheron N, Moore M, O'Brien W, Harris S, Roderick P. Feasibility of detection and intervention for alcohol-related liver disease in the community: the Alcohol and Liver Disease Detection study (ALDDeS). Br J Gen Pract. 2013;63(615):e698-705.
9. Hussain A, Patel PJ, Rhodes F, Srivastava A, Patch D, Rosenberg W. Decompensated cirrhosis is the commonest presentation for NAFLD patients undergoing liver transplant assessment. Clin Med (Lond). 2020;20(3):313-8.
10. Srivastava A, Gailer R, Tanwar S, Trembling P, Parkes J, Rodger A, et al. Prospective evaluation of a primary care referral pathway for patients with non-alcoholic fatty liver disease. J Hepatol. 2019;71(2):371-8.
11. Srivastava A, Jong S, Gola A, Gailer R, Morgan S, Sennett K, et al. Cost-comparison analysis of FIB-4, ELF and fibroscan in community pathways for non-alcoholic fatty liver disease. Bmc Gastroenterology. 2019;19.
12. NICE-Guidance. Cirrhosis in Over 16s: Assessment and Management. National Institute for Health and Care Excellence: Guidance. 2016 [Available from: https://www.nice.org.uk.]

For peer review only - http://bmjopen.bmj.com/site/about/guidelines.xhtml

13. Thiele M, Madsen BS, Hansen JF, Detlefsen S, Antonsen S, Krag A. Accuracy of the Enhanced Liver Fibrosis Test vs FibroTest, Elastography, and Indirect Markers in Detection of Advanced Fibrosis in Patients With Alcoholic Liver Disease. Gastroenterology. 2018;154(5):1369-79.
14. Ekstedt M, Franzen L, M H, Bendtsen P, Mathiesen UL, Bodemar G, et al. Alcohol consumption is associated with progression of hepatic fibrosis in non-alcoholic fatty liver disease. Scand J Gastroenterol. 2009;44(3):366-74.
15. Bataller R, Gao B. Liver fibrosis in alcoholic liver disease. Semin Liver Dis. 2015;35(2):146-56.
16. Becker U, Deis A, Sorensen TI, Gronbaek M, Borch-Johnsen K, Muller CF, et al. Prediction of risk of liver disease by alcohol intake, sex, and age: a prospective population study. Hepatology. 1996;23(5):1025-9.
17. Sanchez-Jimenez BA, Brizuela-Alcantara DC, Ramos-Ostos MH, Alva-lopez F, Uribe-Esquivel M, Chavez-Tapia N. Both alcoholic and non-alcoholic steatohepatitis association with cardiovascular risk and liver fibrosis. Alcohol. 2018;69:63-7.
18. Glyn-Owen K, Bohning D, Parkes J, Roderick P, Buchanan R. The combined effect of alcohol and body mass index on risk of chronic liver disease: A systematic review and meta-analysis of cohort studies. Liver Int. 2020.
19. Glyn-Owen K, Bohning D, Parkes J, Roderick P, Buchanan R. The combined effect of alcohol and obesity on risk of liver disease: a systematic review and meta-analysis. . Hepatology. 2019;70(S1):753A-A.
20. Nguyen-Khac E, Chatelain D, Tramier B, Decrombecque C, Robert B, Joly JP, et al. Assessment of asymptomatic liver fibrosis in alcoholic patients using fibroscan: Prospective comparison with seven non-invasive laboratory tests. Alimentary Pharmacology and Therapeutics. 2008;28(10):1188-98.
21. Chan WC, Treeprasertsuk S, Goh BBG, al. e. Optimising use of nonalcoholic fatty liver disease fibrosis score, Fibrosis-4 score, and liver stiffness measurement to identify patients with advanced fibrosis. Clin Gastroenterol Hepatol. 2019;17:2570-80.
22. Alberti KG, Eckel RH, Grundy SM, Zimmet PZ, Cleeman JI, Donato KA, et al. Harmonizing the metabolic syndrome: a joint interim statement of the International Diabetes Federation Task Force on Epidemiology and Prevention; National Heart, Lung, and Blood Institute; American Heart Association; World Heart Federation; International Atherosclerosis Society; and International Association for the Study of Obesity. Circulation. 2009;120(16):1640-5.
23. Moreno C, Mueller S, Szabo G. Non-invasive diagnosis and biomarkers in alcohol-related liver disease. J Hepatol. 2019;70(2):273-83.
24. Hagstrom H, Nasr P, Ekstedt M, Kechagias S, Onnerhag K, Nilsson E, et al. Low to moderate lifetime alcohol consumption is associated with less advanced stages of fibrosis in non-alcoholic fatty liver disease. Scandinavian Journal of Gastroenterology. 2017;52(2):159-65.
25. Corrao G, Bagnardi V, Zambon A, Arico S. Exploring the dose-response relationship between alcohol consumption and the risk of several alcohol-related conditions: a meta-analysis. Addiction. 1999;94(10):1551-73.
26. Corrao G, Bagnardi V, Zambon A, La Vecchia C. A meta-analysis of alcohol consumption and the risk of 15 diseases. Prev Med. 2004;38(5):613-9.
27. Corrao G, Bagnardi V, Zambon A, Torchio P. Meta-analysis of alcohol intake in relation to risk of liver cirrhosis. Alcohol Alcohol. 1998;33(4):381-92.

For peer review only - http://bmjopen.bmj.com/site/about/guidelines.xhtml

28. Kamper-Jorgensen M, Gronbaek M, Tolstrup J, Becker U. Alcohol and cirrhosis: dose--response or threshold effect? J Hepatol. 2004;41(1):25-30.
29. Patel PJ, Smith D, Connor JP, Horsfall LU, Hayward KL, Hossain F, et al. Alcohol Consumption in Diabetic Patients with Nonalcoholic Fatty Liver Disease. Can J Gastroenterol Hepatol. 2017;2017:7927685.

For peer review only

For peer review only - http://bmjopen.bmj.com/site/about/guidelines.xhtml

Figure 1: Flow chart depicting reclassification of aetiologies

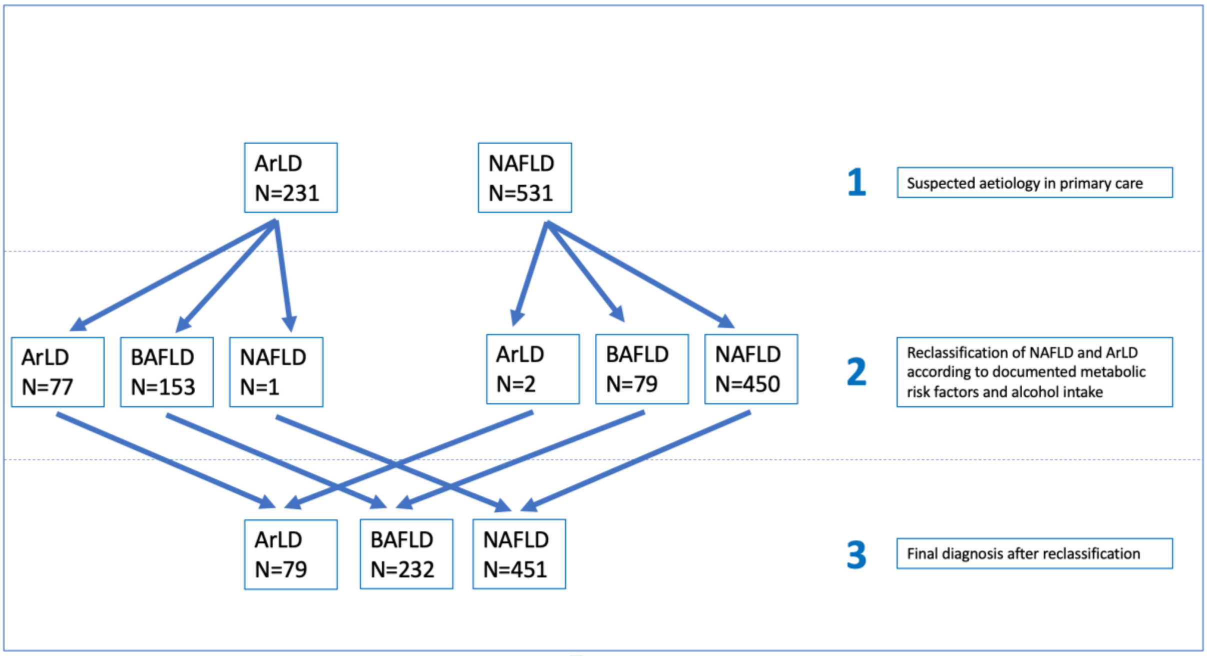

For peer review only - http://bmjopen.bmj.com/site/about/guidelines.xhtml

Figure 2: ROC analysis of the performance of indirect tests for fibrosis and simple liver blood tests in the detection of advanced fibrosis (composite clinical judgement) in patients referred with suspected ArLD. (N=231)

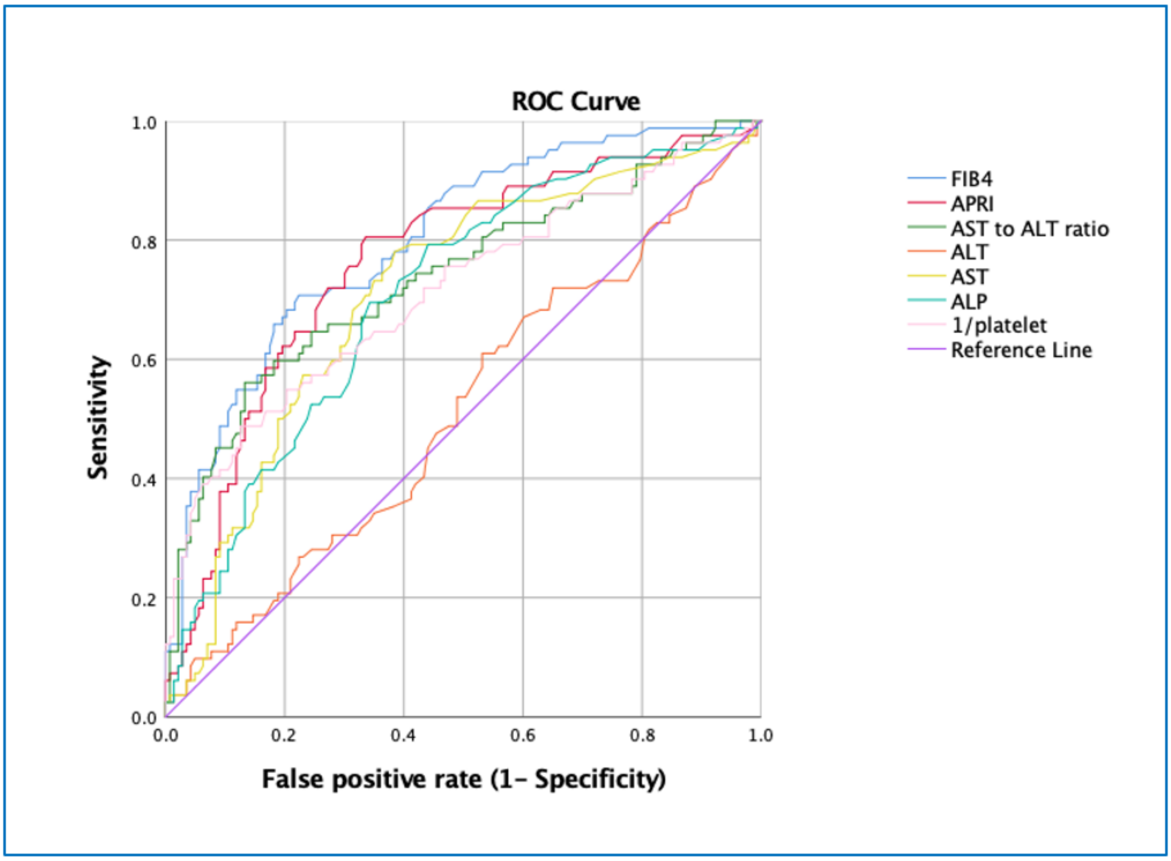

AUROCs with 95% CI in brackets: FIB4: 0.801 (0.742 to 0.860); APRI: 0.763 (0.697 to 0.829); AST:ALT ratio: 0.739 (0.668 to 0.809); ALT: 0.512 (0.433 to 0.591); AST: 0.711 (0.640 to 0.782); ALP: 0.708 (0.638 to 0.777); 1/platelet: 0.714 (0.641 to 0.787). (All p values <0.001 apart from ALT which was non-significant at p = 0.758)

For peer review only - http://bmjopen.bmj.com/site/about/guidelines.xhtml

Supplementary Table 1: Inclusion and exclusion criteria

Inclusion Criteria	Exclusion criteria
○ Age 18 or above	○ Presence of pre-existing hepatological diagnosis (Including but not limited to: auto-immune hepatitis, viral hepatitis, PBC, PSC, HCC),
○ Presence of new referral letter from GP to hepatology clinic at Royal Free during evaluation period Jan 2015 to Jan 2018	○ Patients are already under the care of a hepatologist/Gastroenterologist for investigation or management of a liver condition.
○ Primary reason for referral from GP to hepatologist is suspected diagnosis of ArLD$ or suspected diagnosis of NAFLD^	

$ ‘Suspected ArLD’ referrals were defined as those in which the GP referral letter requested an assessment by a liver specialist specifying concerns about suspected ArLD or expressing concerns about a patient’s alcohol intake.

^ ‘Suspected NAFLD’ referrals were defined as those in which the GP referral letter either specified that they were referring the patient to hepatology ‘with suspected NAFLD’ or ‘on the local NAFLD referral pathway’, OR, in the absence of any other cause of liver dysfunction, where the GP specified that the patient had steatosis or chronic liver disease on ultrasound in combination with mentioning metabolic risk factors (BMI ≥25, diabetes, high waist circumference, high cholesterol or hypertension).

For peer review only

Supplementary table 2: Demographics within each re-classified aetiology group

**Overall characteristics (n=762)**	**ArLD (n =79)**	**NAFLD (n=451)**	**BAFLD (n=232)**	
**Non advanced fibrosis (<F3) n (%)**	46/78 (60)	377/450 (83.8)	165/230 (71.7)	*p<0.001*
**Advanced fibrosis (≥F3) n (%)**	32/78 (40)	73/450 (16.2)	65/230 (28.3)	
**Age (mean; sd)**	51.85 ± 13.1	55.3 ± 14.07	57.2 ± 12.3	*p= 0.009*
**BMI (mean; sd)**	21.9 ± 2.32	32.1± 6.17	30.6 ± 5.03	*p<0.001*
**> 25 n (%)**	0/59 (0)	393/443 (88.7)	215/230 (93.3)	*p<0.001*
**> 30 n (%)**	0/57 (0)	252/416 (60.6)	98/202 (48.5)	*p<0.001*
**Alcohol intake median U/w (IQR)**	79.9 (49.3-140)	0 (0-4)	49.5 (30-88.5)	*p<0.001*
**N=**	76	434	228	
**Years of harmful drinking Median (IQR)**	13 (5-20)	0 (0-0)	20 (8-30)	*p<0.001*
**N=**	47	427	124	
**ALT median, (IQR)**	43 (28-68)	45 (31-68.25)	47 (30-67)	*p=0.752*
**N=**	79	n 450	232	
**Community ELF score (mean, sd)**	9.96 ± 0.42	10.33 ± 0.74	10.5 ± 0.84	*p= 0.215*
**N=**	7	169	54	
**Community FIB4 median (IQR)**	2.75 (1.22-5.19)	1.56 (1.38 -2.2)	2.2 (1.5-3.25)	*p =0.043*
**N=**	4	75	24	

ArLD = Alcohol-related Liver Disease, NAFLD = Non-Alcoholic-Fatty-Liver-Disease, BAFLD = Both Alcohol and Fatty Liver Disease, sd = standard deviation, BMI = Body Mass Index, IQR = interquartile range, ALT = alanine aminotransferase, ELF = Enhanced Liver Fibrosis score

For peer review only

STROBE Statement—Checklist of items that should be included in reports of ***cross-sectional studies***

	Item No	Recommendation	Page No
**Title and abstract**	1	(*a*) Indicate the study's design with a commonly used term in the title or the abstract	1
		(*b*) Provide in the abstract an informative and balanced summary of what was done and what was found	4
**Introduction**			
Background/rationale	2	Explain the scientific background and rationale for the investigation being reported	6
Objectives	3	State specific objectives, including any prespecified hypotheses	7
**Methods**			
Study design	4	Present key elements of study design early in the paper	7
Setting	5	Describe the setting, locations, and relevant dates, including periods of recruitment, exposure, follow-up, and data collection	7
Participants	6	(*a*) Give the eligibility criteria, and the sources and methods of selection of participants	7
Variables	7	Clearly define all outcomes, exposures, predictors, potential confounders, and effect modifiers. Give diagnostic criteria, if applicable	7
Data sources/ measurement	8*	For each variable of interest, give sources of data and details of methods of assessment (measurement). Describe comparability of assessment methods if there is more than one group	7
Bias	9	Describe any efforts to address potential sources of bias	7
Study size	10	Explain how the study size was arrived at	7
Quantitative variables	11	Explain how quantitative variables were handled in the analyses. If applicable, describe which groupings were chosen and why	8
Statistical methods	12	(*a*) Describe all statistical methods, including those used to control for confounding	8
		(*b*) Describe any methods used to examine subgroups and interactions	8
		(*c*) Explain how missing data were addressed	n/a
		(*d*) If applicable, describe analytical methods taking account of sampling strategy	n/a
		(*e*) Describe any sensitivity analyses	9
**Results**			
Participants	13*	(a) Report numbers of individuals at each stage of study—eg numbers potentially eligible, examined for eligibility, confirmed eligible, included in the study, completing follow-up, and analysed	10
		(b) Give reasons for non-participation at each stage	n/a
		(c) Consider use of a flow diagram	Fig1
Descriptive data	14*	(a) Give characteristics of study participants (eg demographic, clinical, social) and information on exposures and potential confounders	10
		(b) Indicate number of participants with missing data for each variable of interest	11
Outcome data	15*	Report numbers of outcome events or summary measures	12
Main results	16	(*a*) Give unadjusted estimates and, if applicable, confounder-adjusted estimates and their precision (eg, 95% confidence interval). Make clear which confounders were adjusted for and why they were included	12

For peer review only

		(*b*) Report category boundaries when continuous variables were categorized	13
		(*c*) If relevant, consider translating estimates of relative risk into absolute risk for a meaningful time period	n/a
Other analyses	17	Report other analyses done—eg analyses of subgroups and interactions, and sensitivity analyses	14
**Discussion**			
Key results	18	Summarise key results with reference to study objectives	15
Limitations	19	Discuss limitations of the study, taking into account sources of potential bias or imprecision. Discuss both direction and magnitude of any potential bias	17
Interpretation	20	Give a cautious overall interpretation of results considering objectives, limitations, multiplicity of analyses, results from similar studies, and other relevant evidence	17
Generalisability	21	Discuss the generalisability (external validity) of the study results	17
**Other information**			
Funding	22	Give the source of funding and the role of the funders for the present study and, if applicable, for the original study on which the present article is based	3

*Give information separately for exposed and unexposed groups.

Note: An Explanation and Elaboration article discusses each checklist item and gives methodological background and published examples of transparent reporting. The STROBE checklist is best used in conjunction with this article (freely available on the Web sites of PLoS Medicine at http://www.plosmedicine.org/, Annals of Internal Medicine at http://www.annals.org/, and Epidemiology at http://www.epidem.com/). Information on the STROBE Initiative is available at www.strobe-statement.org.

For peer review only
